# ACPA Alleviates Bleomycin-Induced Pulmonary Fibrosis by Inhibiting TGF-β-Smad2/3 Signaling-Mediated Lung Fibroblast Activation

**DOI:** 10.3389/fphar.2022.835979

**Published:** 2022-03-09

**Authors:** Dongxin Chen, Huirong Tang, Hongchao Jiang, Lei Sun, Wenjuan Zhao, Feng Qian

**Affiliations:** ^1^ Engineering Research Center of Cell and Therapeutic Antibody, Ministry of Education, School of Pharmacy, Shanghai Jiao Tong University, Shanghai, China; ^2^ Anhui Province Key Laboratory of Translational Cancer Research, Bengbu Medical College, Bengbu, China

**Keywords:** arachidonoylcyclopropylamide, cannabinoid type 1 receptor, fibroblast, idiopathic pulmonary fibrosis, transforming growth factor-beta

## Abstract

Pulmonary fibrosis is a group of life-threatening diseases with limited therapeutic options. The involvement of cannabinoid type 1 receptors (CB_1_R) has been indicated in fibrotic diseases, but whether or not the activation of CB_1_R can be a benefit for fibrosis treatment is controversial. In this study, we investigated the effects of arachidonoylcyclopropylamide (ACPA), as a selective CB_1_R agonist, on bleomycin (BLM)-induced pulmonary fibrosis. We showed that ACPA treatment significantly improved the survival rate of BLM-treated mice, alleviated BLM-induced pulmonary fibrosis, and inhibited the expressions of extracellular matrix (ECM) markers, such as collagen, fibronectin, and α-SMA. The enhanced expressions of ECM markers in transforming growth factor-beta (TGF-β)-challenged primary lung fibroblasts isolated from mouse lung tissues were inhibited by ACPA treatment in a dose-dependent manner, and the fibroblast migration triggered by TGF-β was dose-dependently diminished after ACPA administration. Moreover, the increased mRNA levels of CB_1_R were observed in both lung fibroblasts of BLM-induced fibrotic mice *in vivo* and TGF-β-challenged primary lung fibroblasts *in vitro*. CB_1_R-specific agonist ACPA significantly diminished the activation of TGF-β–Smad2/3 signaling, i.e., the levels of p-Smad2 and p-Smad3, and decreased the expressions of downstream effector proteins including slug and snail, which regulate ECM production, in TGF-β-challenged primary lung fibroblasts. Collectively, these findings demonstrated that CB_1_R-specific agonist ACPA exhibited antifibrotic efficacy in both *in vitro* and *in vivo* models of pulmonary fibrosis, revealing a novel anti-fibrosis approach to fibroblast-selective inhibition of TGF-β-Smad2/3 signaling by targeting CB_1_R.

## Introduction

Pulmonary fibrosis is a group of chronic, progressive, and usually lethal lung diseases that can be idiopathic or secondary to various diseases, characterized by excessive production and massive deposition of extracellular matrix (ECM) in lung interstitium (Barratt et al., 2018). Idiopathic pulmonary fibrosis (IPF) is a severe type of pulmonary fibrosis with unknown etiology and high mortality. However, available therapeutic options for pulmonary fibrosis, including two FDA‐approved IPF drugs (nintedanib and pirfenidone) that slow the progression of the disease, have barely improved the outcomes of patients (Trachalaki et al., 2021). Currently, the prognosis for patients with pulmonary fibrosis remains poor, and it is urgent to identify therapeutic targets and to search novel therapies to slow down or even halt the progression of the disease.

Fibroblasts are the primary cells that build and maintain the ECM. In the pathogenesis of pulmonary fibrosis, lung fibroblasts are activated under the stimulation of profibrotic cytokines and exhibit a series of cell behavior changes, such as proliferation, migration, and ECM production, which is associated with severity of the disease in patients with pulmonary fibrosis and mouse models of pulmonary fibrosis (Xia et al., 2014; DePianto et al., 2015; Kasam et al., 2020). TGF-β family proteins, especially TGF-β1, released by injured lung epitheliums, immune cells, and fibrocytes during pulmonary fibrosis, are considered the principal profibrotic cytokines that drives fibrotic responses (Fernandez and Eickelberg, 2012; Martinez et al., 2017; Aschner et al., 2020; Lee et al., 2020). TGF-β signals are generally transduced through TGF-β type I and type II receptors (TβRI and TβRII) and Smads, and ancillary proteins as well (Aschner et al., 2020; Lee et al., 2020). Although TGF-β signals are attractive targets for lung fibrosis, genetic and pharmacological studies have indicated that broad targeting of general TGF-β signaling pathways might be problematic for treating fibrotic disease due to the pleiotropic roles of TGF-β (Mora et al., 2017; Saito et al., 2018). Therefore, the indirect modulation of TGF-β signals might be an alternative option for therapy of pulmonary fibrosis.

The endocannabinoid system (ECS) plays a critical homeostatic role in the regulation of various cellular and physiological processes through the activation of cannabinoid receptors, and its dysregulation is implicated to contribute to several highly prevalent diseases and disorders (Pacher and Kunos, 2013; Iannotti et al., 2016; Zhou et al., 2021). ECS has demonstrated anti-fibrotic effects in an avalanche of experimental studies, and cannabinoids are considered to be promising for the treatment of fibrosis (Correia-Sá et al., 2020a; Correia-Sá et al., 2020b). However, there is some controversy regarding the cannabinoid system’s effects on fibrosis, especially regarding the cannabinoid type 1 receptors (CB_1_R), a most well-researched cannabinoid receptor that has been reported overexpressed and activated widely in multiple fibrotic processes (Garcia-Gonzalez et al., 2009; Lecru et al., 2015; Cinar et al., 2017). CB_1_R has been found to be associated with multiple pathologic processes in fibrosis, such as promoting/suppressing inflammatory responses, e.g., leukocyte infiltration, microphage activation and cytokine production, in addition to promoting/alleviating fibroblast activation and collagen accumulation in fibrosis (Garcia-Gonzalez et al., 2009; Marquart et al., 2010; Lazzerini et al., 2012; Bronova et al., 2015; Lecru et al., 2015; Chiurchiù et al., 2016; Cinar et al., 2017; Correia-Sá et al., 2020a; Correia-Sá et al., 2020b). Although Cinar R et al. found that the overactivity of CB1 contributed to the pathogenesis of IPF (Cinar et al., 2017), the CB_1_R inhibitor rimonabant had no significant efficacy in stopping progression of fibrosis or reversing established fibrosis (Cinar et al., 2016), the pathology of liver fibrosis was still evident in CB_1_R knockout mice as well (Teixeira-Clerc et al., 2006; Cinar et al., 2016). Similarly, Wang S et al. recently demonstrated that specific deletion of CB_1_R in hepatic stellate cells did not protect mice from fibrosis (Wang et al., 2021). Instead, cannabinoids, such as Δ^9^-tetrahydrocannabinol (THC), cannabidiol, cannabichromene, cannabinol, Cesamet (nabilone; Meda Pharmaceuticals, Somerset, NJ, United States), Marinol (dronabinol; THC; AbbVie, Inc., North Chicago, IL, USA), and Sativex (Cannabis extract; GW Pharmaceuticals, Cambridge, USA) were considered useful for the treatment of chronic inflammation and fibrosis (Zurier and Burstein, 2016). Therefore, the role of CB1 signaling in the development of fibrosis, including IPF, has not yet been identified. It is necessary to clarify whether the activation of CB_1_R is beneficial or harmful and how it affects the development of pulmonary fibrosis for the pharmaceutical development of CB_1_R agonists or antagonists and their potential therapeutic uses in pulmonary fibrosis.

Arachidonoylcyclopropylamide (ACPA) is a selective CB_1_R agonist, which is a close analog of endocannabinoid anandamide and binds to the CB_1_R with a strong affinity and high selectivity (Hillard et al., 1999). In this work, we explored the efficacy of CB_1_R-specific agonist ACPA on bleomycin (BLM)-induced pulmonary fibrosis mouse models and ECM production in TGF-β1-challenged lung fibroblasts. We also revealed the role of CB_1_R in fibroblast activation in the progression of pulmonary fibrosis, which might be a druggable target for pulmonary fibrosis therapy.

## Materials and Methods

### Reagents

ACPA (N-cyclopropyl-5Z, 8Z, 11Z, 14Z-eicosatetraenamide; C23H37NO; CAS: 229021-64-1; MW: 343.6, purity ≥98%) was obtained from Cayman Chemical Company. BLM (#RB003) was purchased from BioTang (Lexington, MA, USA). Recombinant TGF-β1 protein (#240-B) was manufactured by R&D Systems (Minneapolis, MN, USA). Primary antibodies against α-smooth muscle actin (α-SMA, #19245), Collagen (#72026), Smad2 (#5339), phospho-Smad2 (Ser465/Ser467, #18338), Smad3 (#9523), phospho-Smad3 (Ser423/425, #9520), Slug (#9585), Snail (#3879), and β-actin (#3700) were purchased from Cell Signaling Technology (Danvers, MA, USA). Primary antibodies against CB_1_R (#ab23703), TGF-β receptor Ⅱ, and Fibronectin (#ab2413) were purchased from Abcam Company (Cambridge, MA, USA). Primary antibody against CB_2_R (#sc293188) was from Santa Cruz Biotechnology (Santa Cruz, CA, USA). Anti-rabbit IgG (Alexa Fluor 488 Conjugate) (#4412) was purchased from Cell Signaling Technology (Danvers, MA, USA). DAPI (#C1002), Forskolin (#S1612), Hematoxylin and Eosin (H&E) staining kit (#C0105), and BeyoClick™ EdU Cell Proliferation Kit were purchased from Beyotime (Shanghai, China). Masson staining Kit (#D026-1-3) was obtained from NanJing JianCheng (China). Reverse transcription kit and SYBR Green Real-Time PCR kit were purchased from Toyobo (Osaka, Japan). MTT was purchased from Sigma Chemical Co. (St. Louis, MO, USA).

### Animals and Treatments

Six- to eight-week-old C57BL/6 mice were purchased from the Laboratory Animal Center Shanghai Laboratory Animal Center (SLAC, Shanghai, China). Mice were maintained in a climate-controlled room (25°C, 55% humidity, and 12 h light/darkness cycles), and all procedures were conducted with the use of protocols approved by the Institutional Animal Care and Use Committee at Shanghai Jiao Tong University.

**FIGURE 1:**
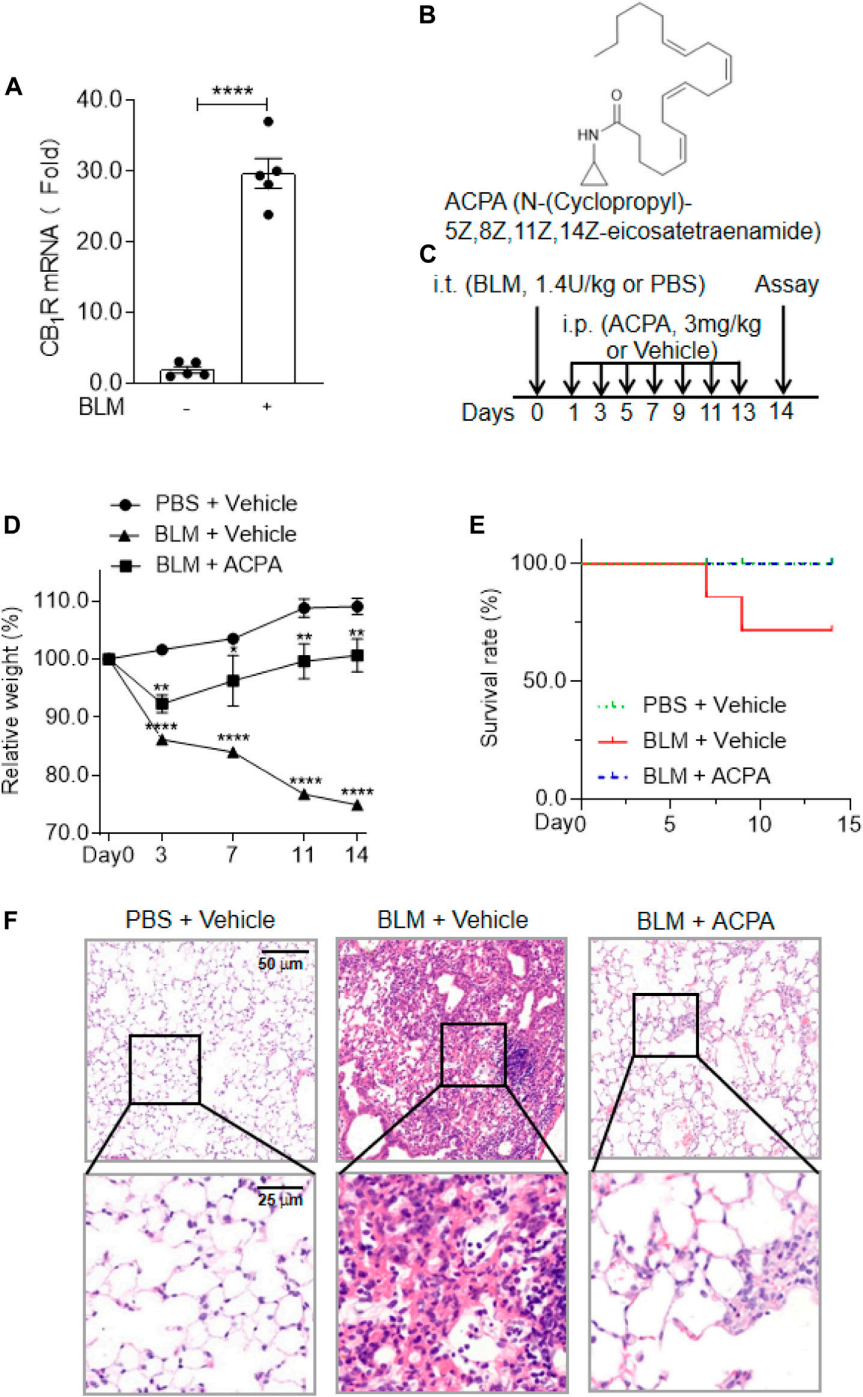
The expression and activation of CB_1_R was beneficial to the mice with bleomycin (BLM)-induced pulmonary fibrosis. **(A)** The mRNA levels of cannabinoid type 1 receptors (CB_1_R) in lung tissues. **(B)** The chemical structure of the selective CB_1_R agonist arachidonoylcyclopropylamide (ACPA). **(C)** The schematic timeline shows the process of BLM model establishment and ACPA administration. C57BL/6 mice were i.t. injected with BLM (1.4 U/kg) or PBS before i.p. injection with ACPA (3 mg/kg) or vehicle, and the mice were sacrificed on day 14. **(D)** Body weight changes in the mice during the experiment. The body weights of the mice were measured at indicated times, and the relative weight was compared with the weight on day 0. **(E)** The survival rate of the mice. **(F)** Representative sections of lung sections performed by hematoxylin and eosin (H&E) staining showing the pathological changes (e.g., infiltration of inflammatory cells and pulmonary lesions). Values are presented as mean ± SEM, n = 5–7 mice per group. *p < 0.05, **p < 0.01, and ***p < 0.001.

C57BL/6 male mice were randomly divided into three groups: the control (PBS + Vehicle) group, BLM (BLM + Vehicle) group, and ACPA (BLM + ACPA) group. ACPA was dissolved in a vehicle (1:1 mixture of PBS and ethanol). For the experiments (Figure 1C), the mice received an intratracheal injection (*i.t.*) of BLM (1.4 U/kg) or PBS at a volume of 20 μl per mouse after anesthetized with pentobarbital sodium (50 mg/kg) by intraperitoneal injection (i.p.). From the next day after BLM injection, the mice in the ACPA group were subjected to ACPA (3 mg/kg, i.p.), and the mice in the control and BLM groups received intraperitoneal injection of vehicle solution (1:1 mixture of PBS and ethanol) at a volume of 10 μl/g per body weight every other day (day 0–13). Animals (n = 7 in each group) were inspected daily in survival study, and death was recorded for each animal. Every animal found dead was necropsied. Surviving animals (n = 7 in control group; n = 5 in BLM group; n = 7 in ACPA group) were euthanized at day 14 to collect tissues. The left lungs from three mice of each group were fixed with polyoxymethylene for histological analysis. The remaining right lungs and lungs from other mice were collected for mRNA, protein, and hydroxyproline (HYP) measurements.

### Tissue Analysis

The left lungs from three mice of each group were fixed with 4 ml of 4% polyoxymethylene for 48 h, followed by dehydration in graded alcohol and embedding in paraffin. Then the paraffin-embedded sections were sliced into 5-μm sections and stained with H&E kit (Beyotime, Shanghai, China) or Masson staining kit (Nanjing Jiancheng Bioengineering Institute, Nanjing, China) according to the instructions of the manufacturer. Stained sections were photographed with an Olympus BX51 microscope (Tokyo, Japan).

### Isolation of Primary Mouse Lung Fibroblasts and Cell Culture

The lungs of the euthanized C57BL/6 mice (6–8 weeks old) were perfused with 10 ml of normal saline, removed immediately into ice-cold Hanks’ balanced salt solutions, chopped finely with scissors, and digested with 2 mg/ml of collagenase IV (Sigma-Aldrich, St. Louis, MO, USA) for 0.5 h. Then after washing with PBS three times, the extracted fibroblasts were cultured in Dulbecco’s modified Eagle’s medium (DMEM, Gibco, MA, USA) supplemented with 10% fetal bovine serum (Sijiqing, Shanghai, China), 1% penicillin and streptomycin (Yishen, Shanghai, China) at 37°C in a 5% CO_2_ incubator for 5 days. The medium was changed every 2 days. For the study of the effects of ACPA on the lung fibroblast *in vitro*, the cultured lung fibroblasts were incubated with serum‐free medium containing PBS or TGF-β1 (20 ng/ml) for 24 h, and treated with vehicle or ACPA 0.5 h before TGF-β1 stimulation.

### Hydroxyproline Assay

The collagen contents in lung tissues were evaluated by HYP assay using an HYP assay kit (Nanjing Jiancheng, Nanjing, China). According to the instructions of the manufacturer, the lung tissues were homogenized in 1 ml of hydrolysate and heated in 1 ml of hydrolysate at 95°C for 20 min, and pH was adjusted from 6.0 to 6.8. The hydroxyproline was detected by incubation with chloramine T and *p*-dimethylaminobenzaldehyde, and the absorbance was measured at 550 nm by a Biotek ELx800 (Winooski, VT, USA).

### RNA Extraction and Quantitative Real Time PCR

RNA samples from lung tissues or cells were extracted using Trizol (Sigma-Aldrich, St Louis, MO, United States). cDNA was produced using qPCR RT Master Mix (TOYOBO, Osaka, Japan) according to the protocol of the manufacturer and used in subsequent real-time qPCR reactions. Quantitative real-time PCR was carried out to determine relative gene expression using the SYBR Green Real-time PCR Master Mix (Toyobo, Osaka, Japan) on StepOne Plus (Thermo Fisher Scientific, Waltham, MA, USA) as specified by the manufacturer. The quantification of target genes was identified by comparing Ct values of each sample normalized with Ct value of glyceraldehyde‐3‐phosphate dehydrogenase (GAPDH), and reaction specificity was verified by melting curve analysis. The primer sequences for target genes in real-time PCR assay were obtained from HuaGene Company (Shanghai, China) as shown below: the primers GAPDH forward 5′-CAT​CAC​TGC​CAC​CCA​GAA​GAC​TG-3′ and reverse 5′-ATG​CCA​GTG​AGC​TTC​CCG​TTC​AG-3′, α‐SMA forward 5′-TGC​TGA​CAG​AGG​CAC​CAC​TGA​A-3′ and reverse 5′-CAG​TTG​TAC​GTC​CAG​AGG​CAT​AG-3′, collagen forward 5′-CCT​CAG​GGT​ATT​GCT​GGA​CAA​C-3′ and reverse 5′- CAG​AAG​GAC​CTT​GTT​TGC​CAG​G-3′, fibronectin forward 5′-CCC​TAT​CTC​TGA​TAC​CGT​TGT​CC-3′ and reverse 5′-TGC​CGC​AAC​TAC​TGT​GAT​TCG​G-3 ′, CB_1_R forward 5′-ATC​GGA​GTC​ACC​AGT​GTG​CTG​T-3′ and reverse 5′-CCT​TGC​CAT​CTT​CTG​AGG​TGT​G-3′.

### Western Blot

Protein samples from lung tissues or cultured cells were lysed and extracted with loading buffer containing protease inhibitor cocktail (Sigma-Aldrich, St. Louis, MO, USA) and phosphatase inhibitor cocktail (Roche Applied Science, USA). Protein concentration was measured using BCA Protein Assay kit (Beyotime, Shanghai, China) according to the protocol of the manufacturer. Protein samples were separated in sodium dodecyl sulfate–polyacrylamide gel electrophoresis (SDS-PAGE) and transferred onto nitrocellulose membranes. The membranes were blocked in 10% bovine serum albumin solution for 2 h at room temperature. Then the membranes were incubated with the appropriate primary antibodies overnight at 4°C, followed by the incubation with conjugated secondary antibodies at room temperature for 2 h. To visualize immune complexes, the membranes were stained using chemiluminescent substrate and imaged by a ChemiDoc MP System (Bio-Rad, MA, USA). The intensity of the protein band was quantified by ImageJ software and normalized to β-actin.

### Scratch Wound Migration Assay

Scratch wound migration assay and Transwell chamber migration assay were performed to detect the migration of primary lung fibroblasts. In scratch wound migration assay, lung fibroblasts were inoculated on six-well plates (1 × 10^6^ cells per well) and cultured with complete culture medium overnight until the cells adhere to the wall. Then the cells were washed three times with PBS and cultured in serum‐free medium. A 200-µl pipet tip was used for scraping the cell monolayer in two straight lines as a cross. The cells were washed three times with serum‐free medium to ensure that no cell debris remained in the scratch areas, and the cells were grown in serum-free medium with PBS + Vehicle, TGF-β1 (20 ng/ml) + Vehicle, or TGF-β1 (20 ng/ml) + ACPA (0, 10, 30 μM) for 24 h. Images were captured with a phase-contrast microscope (Olympus, Tokyo, Japan) and analyzed with ImageJ software.

### Transwell Chamber Migration Assay

Lung fibroblasts (50,000 cells in 200 µl serum‐free medium PBS + Vehicle, TGF-β1 (20 ng/ml) + Vehicle, or TGF-β1 (20 ng/ml) + ACPA (0, 10, 30 μM) were transferred to the upper chambers, and 600 µl of DMEM supplemented with 10% fetal bovine serum was added to the lower chambers. After 24 h of incubation, the medium in the upper chambers was discarded, and the cells that traversed to the reverse face were carefully washed with PBS three times, fixed with the pre-prepared fixative (methanol: acetic acid = 2:1), and then stained with 0.1% crystal violet for 20 min. Pictures were taken with a microscope (Olympus, Tokyo, Japan).

### MTT Cell Viability Assay

Lung fibroblasts (1 × 10^4^ cells per well) plated on 96-well plates were treated with vehicle or ACPA (0, 10, 30, and 100 μM) for 24 h. Then 20 μl MTT solution (5 mg/ml) was added into the wells and incubated for 4 h. The culture supernatant was discarded, and 100 μl of DMSO was added into each well. OD absorbance at 490 nm was measured by a microplate reader (BioTek, Vermont, USA).

### EdU Cell Proliferation Assay

The EdU Cell Proliferation Kit was used to measure cell proliferation. Lung fibroblasts (5 × 10^5^ cells per well) were seeded on 12-well plates and cultured with complete culture medium overnight with the cells adhering to the wall. The cells were pretreated with different concentrations of ACPA (0, 30, 100 µM) for 30 min and then stimulated with TGF-β1 (20 ng/ml) for 24 h. According to the instructions of the manufacturer, fresh DMEM containing EdU (10 µM) was added to the wells. After a 2-h incubation, the EdU medium was discarded, and the fibroblasts were fixed with 4% paraformaldehyde for 15 min and permeabilized with 0.3% Triton X-100 for 15 min. Next, the cells were incubated with Click Reaction solution for 30 min and stained with Hoechst for 10 min. Finally, images were captured using the microscope (Olympus, Tokyo, Japan).

### Immunocytochemistry

Fibroblasts were plated in 12-well plates (3 × 10^5^ cells per well) overnight. The cells were treated with ACPA (30 μM) for 30 min before being challenged with 20 ng/ml of TGF-β1 for 24 h. The cells were fixed with 4% PFA for 15 min, permeabilized with 0.3% Triton X-100 for 15 min, and blocked with 5% BSA for 1 h. Then the cells were incubated with TGF-β receptor Ⅱ primary antibody overnight at 4°C. After washing with PBS three times, cells were incubated with Alexa 488-labeled secondary antibody for 2 h at room temperature, and then cells were stained with DAPI (5 μg/ml) to visualize the cell nuclei. Immunofluorescence images were recorded by a laser-scanning confocal fluorescence microscope (Leica Microsystem, Wetzlar, Germany).

### Statistics Analysis

Statistical analysis was performed using the Prism software (ver. 8.3; GraphPad, San Diego, CA, USA). Comparisons between different groups were analyzed by one-way analysis of variance (ANOVA) followed by Tukey’s test, and Student’s t-test was used to compare differences between two groups. All experiment results were presented as the mean ± SEM. The referred experiment was performed independently for at least three times. Statistical significance was defined as *p < 0.05, **p < 0.01, ***p < 0.001, ****p < 0.0001.

## Results

### Cannabinoid Type 1 Receptors Overexpression and Activation Brought About Beneficial Effects on Mice With Bleomycin-Induced Pulmonary Fibrosis

CB_1_R has been found to be linked to multiple pathologic processes in fibrosis, including pulmonary fibrosis (Garcia-Gonzalez et al., 2009; Marquart et al., 2010; Lazzerini et al., 2012; Bronova et al., 2015; Lecru et al., 2015; Chiurchiù et al., 2016; Cinar et al., 2017; Correia-Sá et al., 2020a; Correia-Sá et al., 2020b), but the actual function of CB_1_R activation in pulmonary fibrosis remains controversial. In the present study, we first examined the expression of CB_1_R in the lung tissues of a murine pulmonary fibrosis model by i.t. injection of BLM (1.4 U/kg). We found that the mRNA levels of CB_1_R significantly increased in the lung tissues of mice with pulmonary fibrosis compared with normal mice (Figure 1A), which indicated that overexpressed CB_1_R might play certain role in the pathologic processes of pulmonary fibrosis. Then we observed the influences of CB_1_R activation on mice with BLM-induced pulmonary fibrosis using the highly selective CB_1_R agonist ACPA (Figure 1B) to stimulate CB_1_R. The murine model of pulmonary fibrosis was established by i.t. injection of BLM, and the function of ACPA for pulmonary fibrosis was evaluated by i.p. injection (Figure 1C). Compared with the mice in normal control group, the pulmonary fibrosis mice subjected to BLM i.t. injection exhibited significantly diminished body weight, an important indicator of pathological severity following BLM challenge, while ACPA treatment largely rescued the weight loss of pulmonary fibrosis mice (Figure 1D). Similarly, the survivorship curve also manifested that ACPA treatment improved the survival rate of pulmonary fibrosis mice (Figure 1E). In addition, HE staining demonstrated extensive inflammatory cell infiltration and severe pulmonary lesions in lung tissues of mice with BLM-induced pulmonary fibrosis, which were apparently reduced by ACPA administration (Figure 1F). Collectively, these data showed that CB_1_R overexpression in lung tissues and activation stimulated by ACPA would help to mitigate pathological changes and improve survival of pulmonary fibrosis mice.

### Arachidonoylcyclopropylamide Ameliorated Bleomycin-Induced Pulmonary Fibrosis *in Vivo*

**FIGURE 2:**
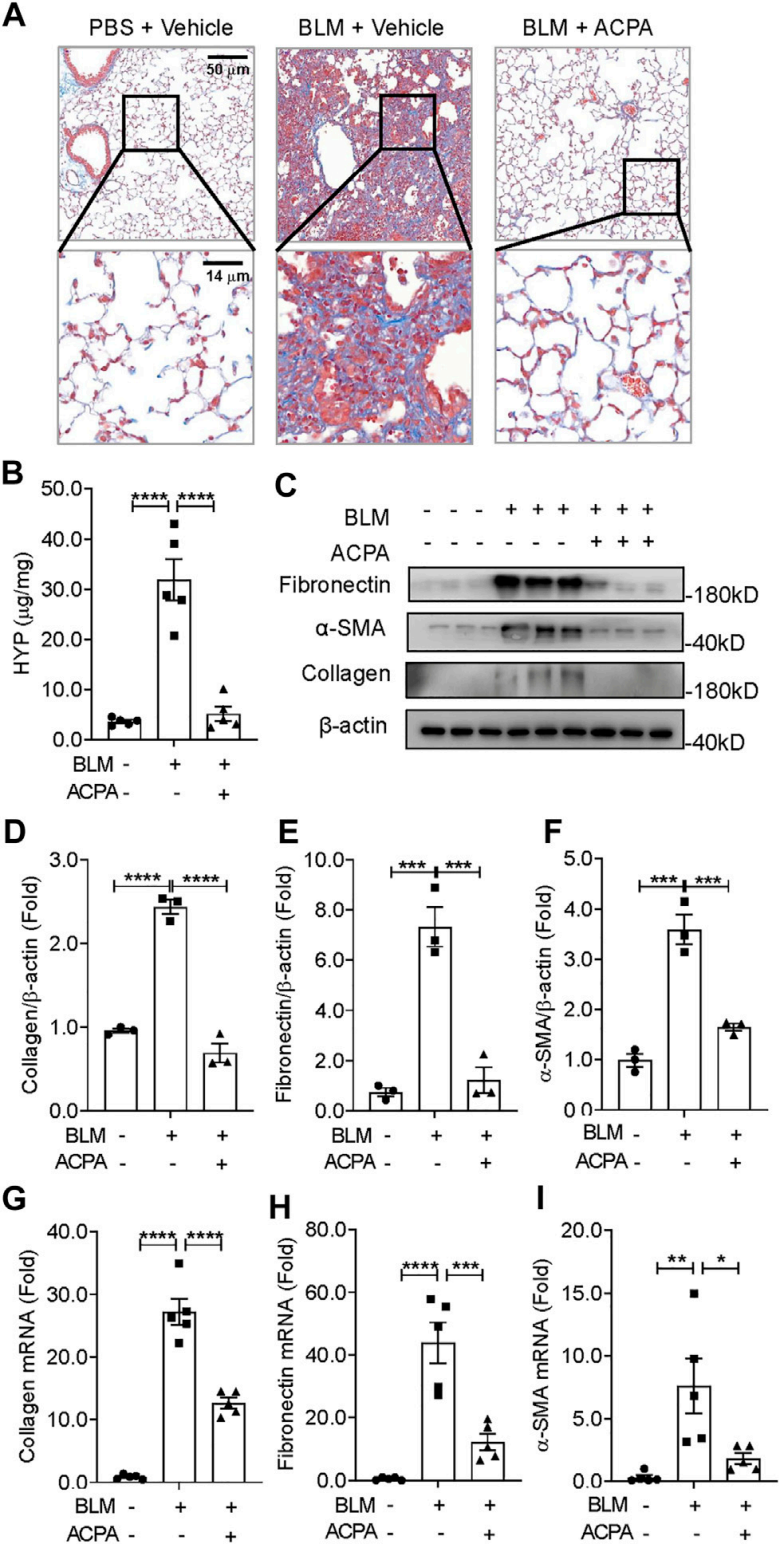
ACPA attenuated BLM-induced pulmonary fibrosis in mice. **(A)** Representative images of lung sections visualized by Masson staining demonstrated collagen deposition, indicative of fibrosis. **(B)** Hydroxyproline (HYP) analysis of lung tissues from mice treated with PBS + Vehicle, BLM + Vehicle, and BLM + ACPA injection. **(C)** Western blots for extracellular matrix (ECM) protein collagen, fibronectin, and α-smooth muscle actin (α-SMA) in lung tissues of mice from each group. Quantification of collagen **(D)**, fibronectin **(E)**, and α-SMA **(F)** proteins normalized to β-actin were analyzed by ImageJ software. Relative mRNA levels of fibrosis markers collagen **(G)**, fibronectin **(H)**, and α-SMA **(I)** in lung tissues of mice from each group were quantified by real-time PCR. Data are presented as means ± SEM, n = 3 per group in **(D–F)** and n = 5 per group in **(B)** and **(G–I)**. *p < 0.05, **p < 0.01, and ***p < 0.001.

Pulmonary fibrosis is a group of lethal disease characterized by deposition of a pathological ECM, we further evaluated the efficacy of CB_1_R-selective agonist ACPA on collagen deposition and the expression of fibrotic makers in BLM-induced experimental pulmonary fibrosis *in vivo* (Figure 2). Histologic assay of Masson staining demonstrated a dramatic collagen accumulation, the indicative of fibrosis, in BLM-stimulated mice, while ACPA treatment strongly attenuated deposition of pulmonary collagen (Figure 2A). Consistently, HYP, the main component of collagen, was also significantly reduced by treatment compared with that in the BLM group (Figure 2B). In line with the results of Masson staining and HYP assay, the productions (protein levels) of ECM, e.g., collagen, fibronectin, and α-SMA, which are biomarkers for fibrotic levels, remarkably decreased in ACPA group (Figures 2C–F). Similarly, the transcription (mRNA levels) of collagen, fibronectin, and α-SMA were also downregulated by ACPA (Figures 2G–I). Collectively, these results showed that CB_1_R-selective agonist ACPA protected pulmonary fibrosis mice against BLM-induced pulmonary fibrosis with the inhibition of ECM production.

### Arachidonoylcyclopropylamide Reduced the Expression of extracellular Matrix Proteins in TGF-β1-Challenged Lung Fibroblasts *in Vitro*

**FIGURE 3:**
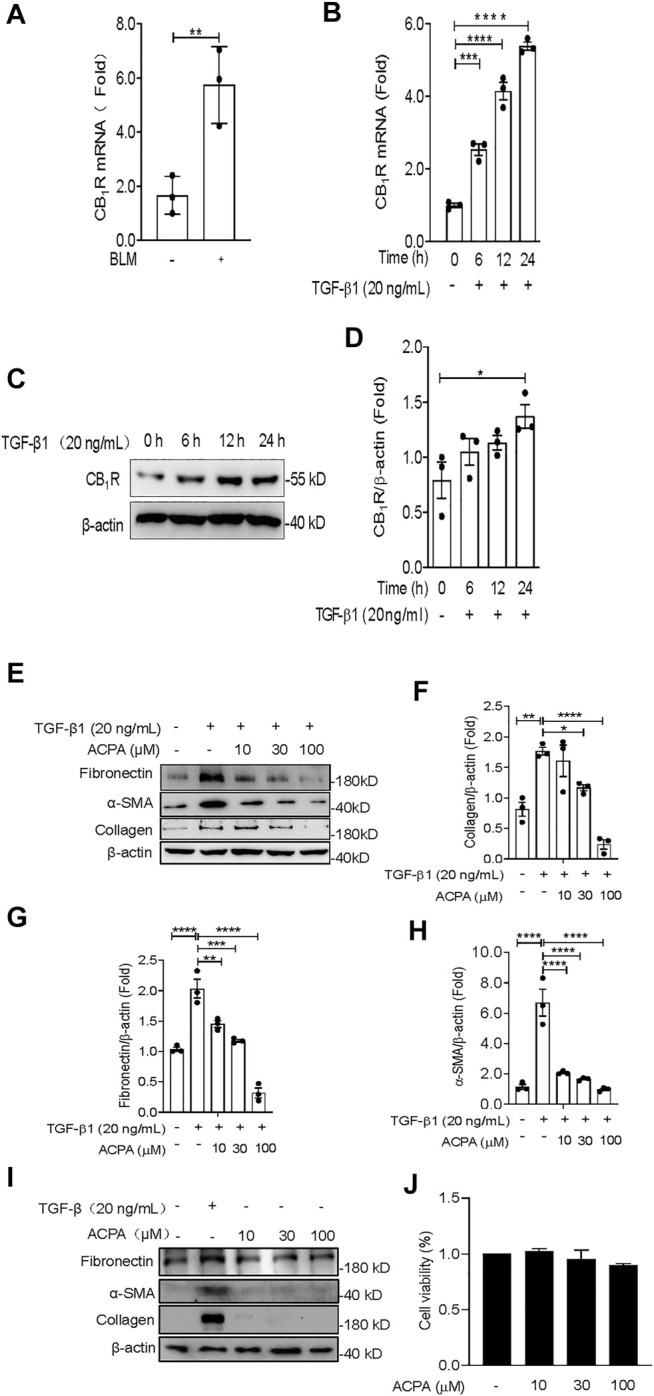
ACPA inhibited the expression of ECM proteins in TGF-β1-challenged primary lung fibroblasts. **(A)** The mRNA levels of CB_1_R in lung fibroblasts from normal control mice or the pulmonary fibrosis model mice treated with BLM (1.4 U/kg, i.t.). **(B)** The mRNA levels of CB_1_R in primary mouse lung fibroblasts pretreated with TGF-β1 (20 ng/ml). **(C)** Western blots for CB_1_R protein in primary mouse lung fibroblasts pretreated with TGF-β1 (20 ng/ml). **(D)** Quantification of CB_1_R proteins normalized to β-actin. **(E)** Western blots for ECM protein collagen, fibronectin, and α-SMA in TGF-β1-challenged primary lung fibroblasts. Primary mouse lung fibroblasts were treated with vehicle or ACPA (10, 30, 100 μM) followed by TGF-β1 stimulation for 24 h. Quantification of collagen **(F)**, fibronectin **(G)**, and α-SMA **(H)** proteins normalized to β-actin were analyzed by ImageJ software. **(I)** Western blots for ECM protein collagen, fibronectin, and α-SMA in primary lung fibroblasts treated with ACPA (10, 30, 100 μM) alone for 24 h. **(J)** The viability of fibroblasts after treatment with ACPA (10, 30, and 100 μM) alone for 24 h in MTT Cell viability assay. Data shown are mean ± SEM from three independent experiments. *p < 0.05, **p < 0.01, and ***p < 0.001.

Fibroblasts are the primary cells that build and maintain the ECM. Increased activation of lung fibroblast is central for the initiation and maintenance of unremitting deposition of ECM and fibrotic lesions in pulmonary fibrosis (Xia et al., 2014; DePianto et al., 2015; Kasam et al., 2020). In this study, we investigated the expression of CB_1_R in lung fibroblasts isolated from BLM-challenged mice and found that CB_1_R mRNA levels were significantly increased in the isolated lung fibroblasts from mice with BLM-induced pulmonary fibrosis compared with control mice (Figure 3A), which is in accordance with the increased CB_1_R mRNA levels in lung tissues of pulmonary fibrosis mice (Figure 1A). TGF-β1 is one of the most potent and well-studied profibrotic inducers of pulmonary fibrosis that triggers and greatly enhances the fibrogenic activity of lung fibroblasts (Fernandez and Eickelberg, 2012; Martinez et al., 2017; Aschner et al., 2020; Lee et al., 2020). We isolated primary mouse lung fibroblasts to examine the expression of CB_1_R and ECM proteins induced by TGF-β1. Primary mouse lung fibroblasts were incubated with PBS or TGF-β1 for 24 h after pretreated with vehicle or ACPA with different concentrations. As shown in Figures 3B–D, both mRNA and protein levels of CB_1_R in primary mouse lung fibroblasts demonstrated a time-dependent augmentation with the stimulation of TGF-β1, suggesting that TGF-β1 promotes CB_1_R expression in lung fibroblasts. By contrast, CB_2_R expression was much lower in lung fibroblasts and its level was not observably changed by TGF-β1 or ACPA treatment (Supplementary Figures S1A–E). Consistent with BLM-induced pulmonary fibrosis, the expression of ECM protein collagen, fibronectin, and α-SMA in primary fibroblasts also increased enormously in response to TGF-β1 stimulation. Although ACPA (10, 30 μM) alone did not affect the expression of ECM proteins (Figure 3I) or exhibit obvious cytotoxicity to fibroblasts (Figure 3J), when administrated with TGF-β1, it significantly reduced the augmentation of ECM proteins induced by TGF-β1, which are also considered as fibrosis markers, in a dose-dependent manner (Figures 3E–H). Taken together, these results revealed that lung fibroblasts expressed CB_1_R when stimulated by TGF-β1 and CB_1_R activation, in turn, inhibiting the excessive expression of ECM proteins in TGF-β-challenged lung fibroblasts.

### Arachidonoylcyclopropylamide Subdued the Migration and Motility of Primary Lung Fibroblasts Triggered by TGF-β1

**FIGURE 4:**
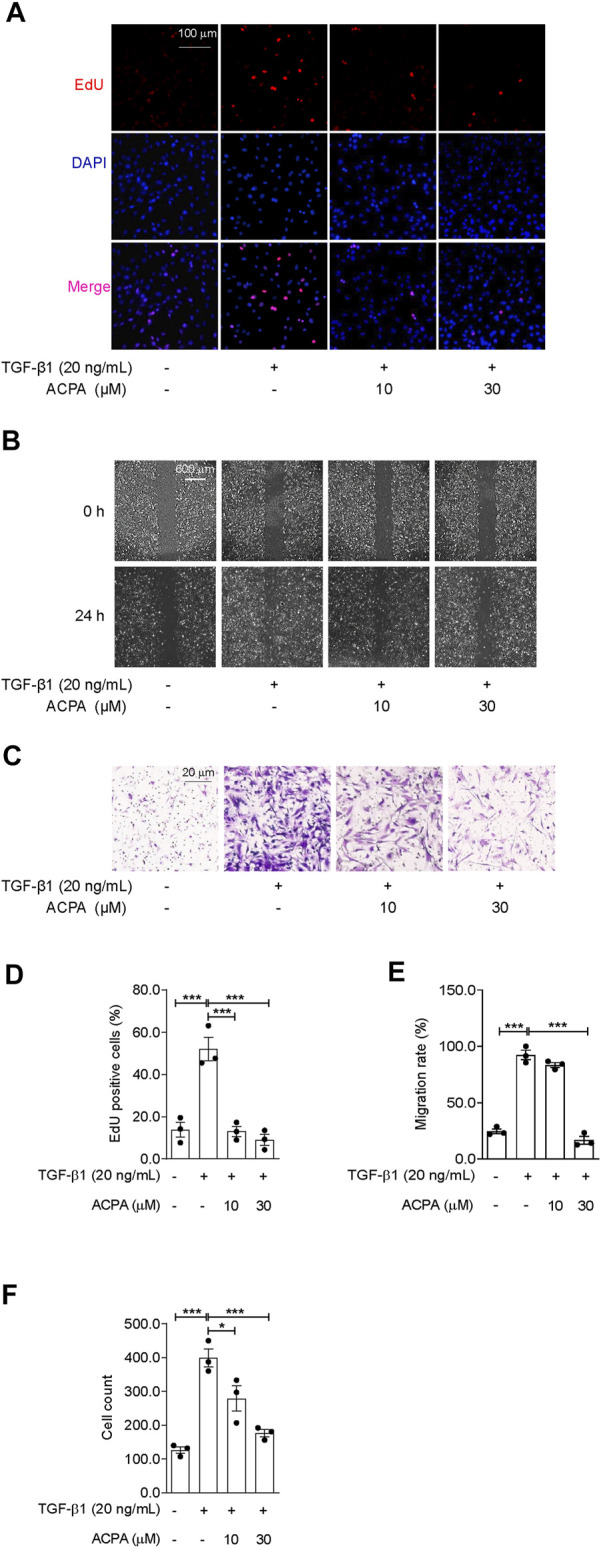
ACPA subdued the cell proliferation and migration of primary lung fibroblasts triggered by TGF-β1. The cell proliferation, migration, and motility of TGF-β1-challenged fibroblasts were detected by EdU cell proliferation assay, scratch wound migration assay, and Transwell migration assay, respectively. Primary mouse lung fibroblasts were pretreated with vehicle or ACPA (10, 30 μM) 0.5 h before TGF-β1 stimulation. **(A)** Representative fluorescence images of EdU staining, nuclei: DAPI (blue), and proliferating cell: EdU + (red). **(B)** Representative images of scratch wounds taken at 24 h after scratch injury in scratch wound migration assay. **(C)** Representative images of the migrated fibroblasts in Transwell migration assay. **(D)** The ratio of proliferating positive cells in EdU cell proliferation assay. **(E)** Quantification of cell migration by measuring the area of the injured region compared with TGF-β1-challenged fibroblasts. **(F)** Cell counting of the migrated fibroblasts in Transwell migration assay. Values are mean ± SEM from three independent experiments; *p < 0.05, **p < 0.01, and ***p < 0.001.

The migratory capacity of fibroblasts by responding to cytokines and chemokines, e.g., TGF-β1, is important for the progression of pulmonary fibrosis (Kasam et al., 2020). To ascertain whether CB_1_R activation participates in modulating TGF-β1-triggered cell migration of primary lung fibroblasts, we conducted scratch wound migration assay and Transwell migration assay on primary lung fibroblasts. Primary fibroblasts were pretreated with CB_1_R-selective agonist ACPA (0, 10, 30 μM) before stimulating with TGF-β1 for 24 h. In EdU cell proliferation assay, TGF-β1 significantly stimulate the proliferation of fibroblasts, as obviously increased EdU-positive cells were seen in TGF-β1 group than those in the control group, and it was reduced by ACPA (10, 30 μM) treatment (Figures 4A, D). Consistently, TGF-β1 significantly spurred fibroblasts to migrate to the scratch guidelines in scratch wound healing assay, while ACPA alleviated the fibroblast migration with the high concentration (30 μM) of ACPA showing a nearly radical inhibition (Figures 4B, E). Moreover, numbers of transmigrating fibroblasts were much higher among TGF-β1-challenged fibroblasts than controls, while ACPA (30 μM) obviously decreased the migration of the lung fibroblasts toward fetal bovine serum (Figures 4C, F). Thus, we concluded that CB_1_R-selective agonist ACPA restrained cell proliferation, migration, and motility of lung fibroblasts triggered by TGF-β1.

### Arachidonoylcyclopropylamide Downregulated TGF-β-Smad2/3 Signaling and the Expression of Snail and Slug in TGF-β1-Challenged Lung Fibroblasts

**FIGURE 5:**
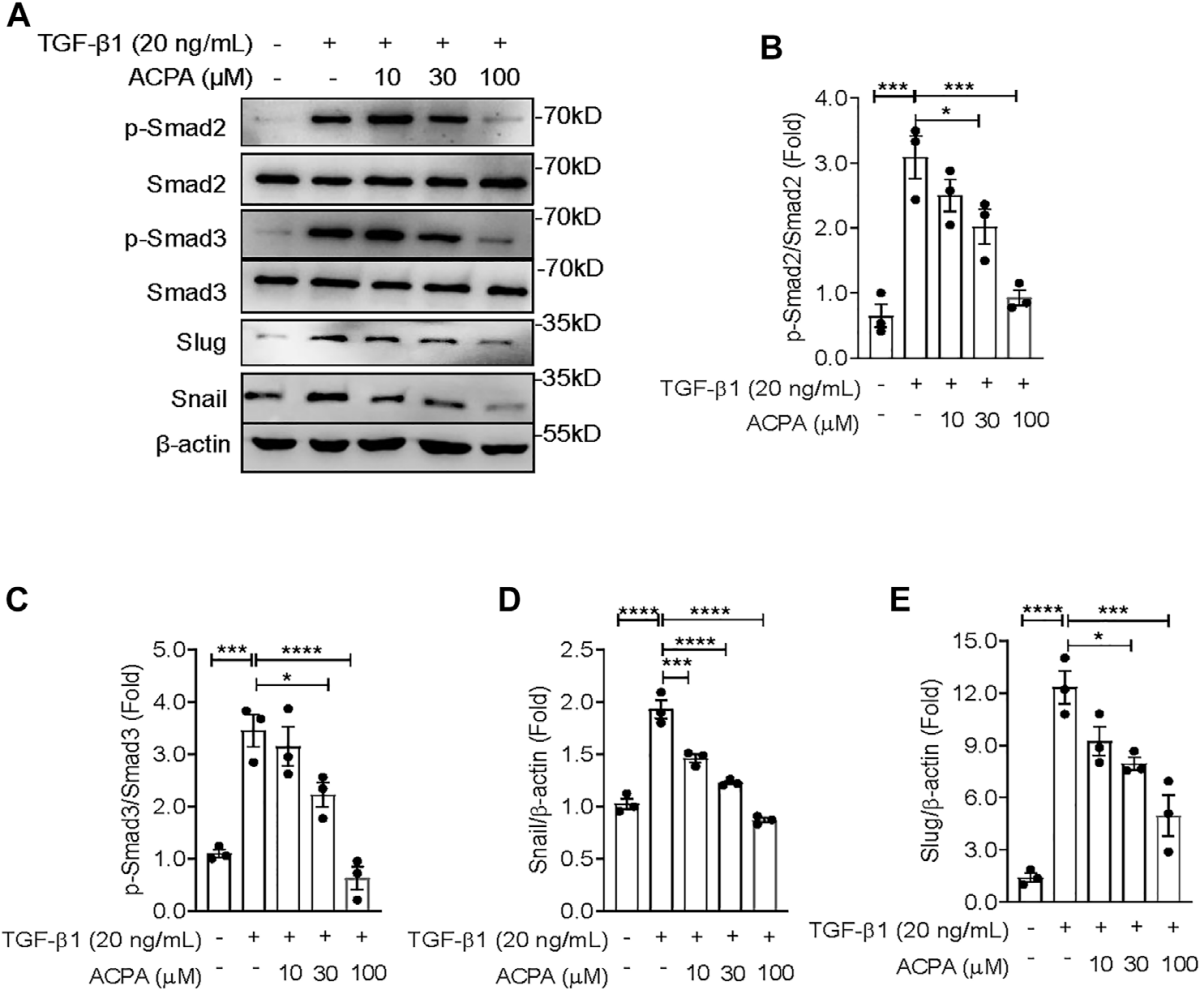
ACPA downregulated the activation of TGF-β-Smad2/3 signaling and the expression of downstream effectors snail and slug in TGF-β1-stimulated lung fibroblasts. **(A)** Western blots for p-Smad2, Smad2, p-Smad3, Smad3, slug, and snail in TGF-β1-challenged lung fibroblasts. Primary mouse lung fibroblasts were treated with vehicle or ACPA (10, 30, 100 μM) followed by TGF-β1 stimulation for 24 h. Quantification of p-Smad2 to Smad2 **(B)**, p-Smad3 to Smad3 **(C)**, snail **(D)**, and slug **(E)** bands normalized to β-actin was pooled from three independent experiments. Data are represented as mean ± SEM. *p < 0.05, **p < 0.01, and ***p < 0.001.

In the present study, we revealed that CB_1_R-selective agonist ACPA substantially suppressed ECM production both in the lung tissues of mice with pulmonary fibrosis and in TGF-β-challenged lung fibroblasts, indicating that CB_1_R activation downregulated TGF-β signaling mediated the expression of ECM proteins in pulmonary fibrosis. To manifest this, we investigated the activation of canonical TGF-β-Smad2/3 signaling and the levels of its downstream effectors in TGF-β1-challenged primary lung fibroblasts with ACPA treatment. Western blot showing that TGF-β1 stimulation resulted in the activation of canonical TGF-β-Smad2/3 signaling, assessed by the elevated p-Smad2 and p-Smad3 (Figures 5A–C), followed by the expression of downstream transcription factors Snail and Slug (Figures 5A, D, E), key regulators that mediated TGF-β-Smad signaling triggered the overexpression and excessive production of ECM proteins in pulmonary fibrosis. However, CB_1_R-selective agonist ACPA exerted a notable inhibition on elevated p-Smad2, p-Smad3, snail, and slug in lung fibroblasts spurred by TGF-β1 (Figure 5). These data indicate that CB_1_R activation acted as a negative regulation of canonical TGF-β-Smad2/3 signaling, downregulated TGF-β triggered Snail and Slug expression, and subsequent overexpression of ECM proteins in lung fibroblasts.

## Discussion

**FIGURE 6:**
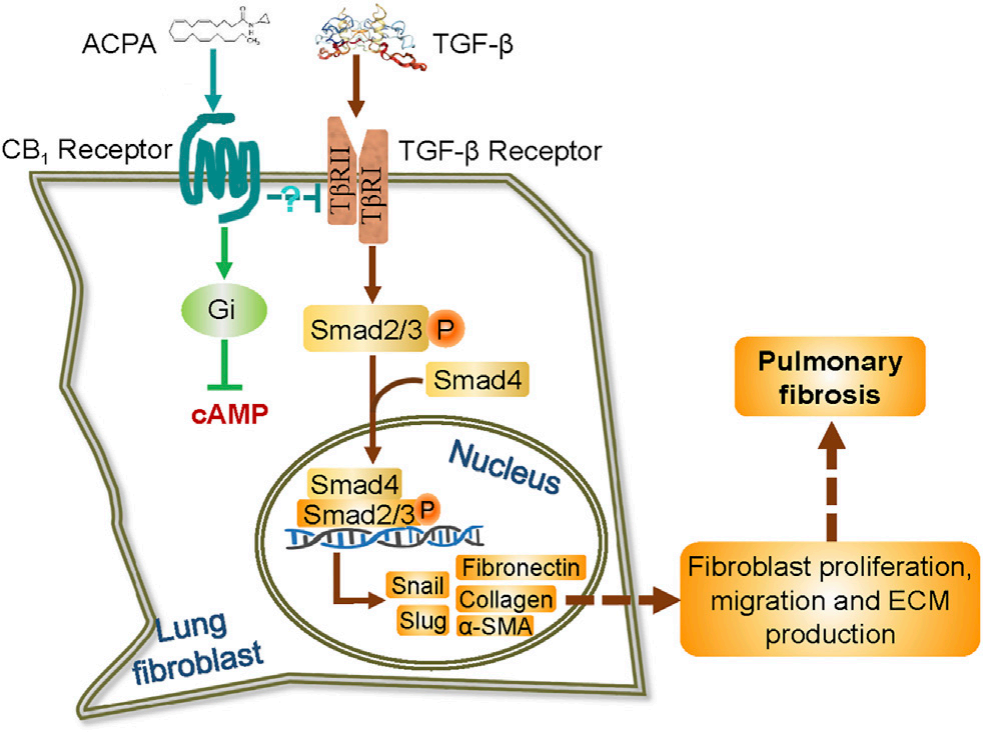
Schematic representation of the possible mechanism by which ACPA inhibits pulmonary fibrosis *via* TGF-β-Smad2/3 signaling-mediated lung fibroblast activation. TGF-β signals are transduced by TGF-β receptor, a TβRI, and TβRII heterodimeric receptor. TGF-β binding to and activating the TGF-β receptor results in the phosphorylation of transcription factors Smad2 and Smad3. The phosphorylated Smad2 and Smad3 then combine with Smad4 in the cytoplasm, and translocate to the nucleus to induce gene transcription, including transcription factor snail and slug and the subsequent unremitting expression of ECM proteins. ACPA selectively binds to and activates CB_1_R of lung fibroblasts, which downregulates TGF-β–Smad2/3 signaling and lead to blockage of ECM production/deposition triggered by TGF-β, by an unknown Gi signaling-independent way.

Pulmonary fibrosis is a life-threatening disease characterized by massive deposition of ECM in the lungs with hardly any therapeutic options. Using both *in vitro* and *in vivo* models of pulmonary fibrosis, we demonstrated that the expression of CB_1_R increased in lung fibroblasts in response to pulmonary fibrosis, and the pharmacologic activation of CB_1_R with its specific agonist ACPA protected against BLM-induced pulmonary fibrosis, significantly decreasing lung fibroblast migration and the excessive expression of ECM proteins (collagen, fibronectin, and α-SMA) stimulated by BLM *in vivo* or TGF-β1 *in vitro*, rather than the basal expression of ECM proteins in normal control lung fibroblasts. Moreover, we demonstrated that CB_1_R-selective agonist ACPA dose-dependently downregulated the activation of TGF-β-Smad2/3 signaling (reduced p-Smad2 and p-Smad3 levels) and the levels of its downstream effectors snail and slug, which are the transcription factors to regulate the expression of ECM proteins. These findings provided the first evidence that CB_1_R acted as a negative regulator for TGF-β1-Smad2/3 signaling and its associated fibroblast activation in pulmonary fibrosis (Figure 6), and thus, the overexpression of CB_1_R on fibroblasts might be a druggable target for the therapy of pulmonary fibrosis.

ECS is an evolutionarily conserved network of signaling systems comprising receptors (such as CB1, CB2, or TRPV-1), their endogenous lipid ligands, or endocannabinoids and synthetic and metabolizing enzymes, present nearly everywhere in the human body. ECS is deeply involved in the maintenance of bodily homeostasis by modulating a wide variety of physiological/pathological processes all over the body (Pacher and Kunos, 2013; Iannotti et al., 2016; Zhou et al., 2021). This is achieved through a negative feedback loop, which works by the activation of synthesis and release of endocannabinoids as they target cannabinoid receptors (Iannotti et al., 2016; Zhou et al., 2021). Increased activity of the endocannabinoid/CB_1_R system was also reported to be parallel with the pathogenesis of pulmonary fibrosis and associated with the increased tissue levels of interferon regulatory factor-5, and the pathogenesis of pulmonary fibrosis, while CB_1_R inhibition, especially the combined CB_1_R/iNOS inhibition, has antifibrotic efficacy in bleomycin-induced pulmonary fibrosis (Cinar et al., 2017). Numerous evidence have demonstrated that CB_1_R was increased and got involved in the progression of multiple organ fibrosis, including the liver (Patsenker et al., 2011), kidneys (Lecru et al., 2015), heart (Slavic et al., 2013), skin (Lazzerini et al., 2012; Garcia-Gonzalez et al., 2016; Cinar et al., 2017), and pulmonary fibrosis as well (Bronova et al., 2015; Cinar et al., 2017). Although the CB_1_R was consistently found highly activated in the pathogenesis of pulmonary fibrosis, the role of activated CB_1_R in fibrosis is controversial. Correia-Sá et al. found that CB_1_R agonist ACEA increases, and antagonist AM251 reduces, collagen deposition induced by TGF-β in the fibroblasts obtained from abdominal human skin (Correia-Sá et al., 2021). However, it has been recently proposed that cannabinoids, natural CB_1_R agonists, may manifest as profibrotic or antifibrotic agents in skin fibrosis (Pryimak et al., 2021). Moreover, CB_1_R inhibitor rimonabant or the deletion of CB_1_R did not prevent, limit, or reverse fibrosis in a substantial body of studies (Teixeira-Clerc et al., 2006; Cinar et al., 2016; Wang et al., 2021). Although the overactivity of CB_1_R was found contributing to the pathogenesis of pulmonary fibrosis, the pharmaceutical development of CB_1_R antagonist and their potential therapeutic uses in fibrosis was halted (Cinar et al., 2016). On the other hand, the phytocannabinoids (THC, cannabidiol, cannabichromene, and cannabinol) and synthetic preparations (Cesamet, Marinol, and Sativex) are candidates for development as anti-inflammatory and antifibrotic agents (Zurier and Burstein, 2016). In the present study, we found that CB_1_R expression dramatically increased in lung tissues and fibroblasts in response to experimental pulmonary fibrosis, but showed that its selective agonist ACPA exhibited marked antifibrotic effect both *in vitro* and *in vivo* models of pulmonary fibrosis, which was inconsistent with CB_1_R inhibition that ameliorated fibrosis (Bronova et al., 2015; Cinar et al., 2017; Correia-Sá et al., 2021). This inconsistency is due to different tissues (lungs, liver, kidneys, or skin), cell types (macrophages *vs.* fibroblasts), pathologic stages of fibrotic diseases (inflammatory stage *vs.* fibrosis stage), and various types of G-protein signaling triggered by CB_1_R. Previous studies have paid close attention to inflammation period and macrophage functions in pulmonary fibrosis, and the inhibition of CB_1_R exhibited anti-inflammatory properties (Cinar et al., 2017). While CB_2_R is expressed primarily in spleen and immune system cells, it had been demonstrated that CB_2_R agonists can be used to treat inflammation-related diseases. In the supplemental experiments, we showed that CB_2_R expression was much lower in lung fibroblasts, and its level was not observably changed by TGF-β1 or ACPA treatment, and the CB_2_R mRNA levels in lung fibroblasts from BLM-challenged mice were increased but with no significant differences, indicating that CB_2_R in lung fibroblasts might not be involved in the inhibition of ACPA on TGF-β1-stimulated fibroblast activation. However, fibroblasts are responsible for mediating the synthesis and deposition of ECM and deteriorating fibrosis in pulmonary fibrosis. It was shown that CB_1_R in mononuclear cells was involved in inflammation, while CB_1_R in fibroblastic cells contributed to wound healing (Zhao et al., 2010). So, the modulation of fibroblast CB_1_R on ECM production might have more impact on fibrosis.

CB_1_R primarily activates Gi/o proteins, which cause downstream inhibition of cAMP accumulation mediated *via* the pertussis toxin-sensitive Gi α-subunit inhibition of adenylyl cyclase. In the supplemental experiments, we used forskolin, a potent adenylate cyclase activator to generate the second messenger, cAMP, to detect the involvement of PKA/cAMP signaling pathway in ACPA-induced downregulation on TGF-β-induced fibroblast activation and found that forskolin did not reverse the inhibition of ACPA on TGF-β1-stimulated ECM production (collagen and α-SMA) of fibroblast (Supplementary Figures S2A, B). Although, the activation of Gi subunit triggered by CB_1_R was shown to have some limit on uncontrolled fibrosis processes (Garcia-Gonzalez et al., 2009), our results suggest that it might not *via* PKA/cAMP signaling pathway and that ACPA downregulates TGF-β1 signaling and inhibits ECM production by fibroblasts (Figure 6). In addition to Gi/o proteins, CB_1_R can also couple to Gs, Gq, and G12/13 α-subunits and Gi βγ-subunit depending on the cellular/protein context, which should not be ruled out. Different agonists can also elicit different patterns of G-protein coupling, e.g., WIN55212-2, but not Δ9-THC and ACEA, is able to stimulate G12/13 in mouse cortex (Diez-Alarcia et al., 2016). Like many other GPCRs, CB_1_R is internalized, which generally occurs as a result of intensive stimulation by an agonist, subsequent receptor phosphorylation, and β-arrestin binding, followed by clathrin-mediated endocytosis (Fletcher-Jones et al., 2020). Binding of β-arrestin 2 to the type III TGF-β receptor (TβRIII) was also triggered by phosphorylation of the receptor mediated by the type II TGF-β receptor (TβRII), which is itself a kinase like GRK, and leads to internalization of both receptors and downregulation of TGF-β signaling (Chen et al., 2003). Thus, the internalization of ACPA-activated CB_1_R and TGF-β1-activated TGF-β receptors might be intertwined, and ACPA might downregulate TGF-β signaling by promoting the endocytosis of TGF-β receptors. This was partly supported by our immunofluorescence analysis on TGF-β receptors located on the cell membrane surface. Our preliminary results showed that TβRII was present predominantly on the cell surface in TGF-β1 (20 ng/ml)-stimulated fibroblasts, and it significantly decreased with ACPA (30 μM) administration together with TGF-β1 (Supplementary Figure S3). Nevertheless, direct evidence that CB_1_R regulated the endocytosis of TGF-β receptors is needed.

The unclear mechanism and etiology render the current therapy, targeting the pulmonary fibrosis, hardly receiving positive feedback among patients. The activation of lung fibroblasts and their accumulation of excessive ECM proteins is essential to develop pulmonary fibrosis and associated with severity of the disease in patients with pulmonary fibrosis (Kasam et al., 2020). Thus, the profibrotic factors in lung fibroblasts is considered an important therapeutic target to develop novel and effective therapies against pulmonary fibrosis. Actually, nintedanib, one of the two approved drugs for fibrotic disease, has been shown to impact signaling pathways of fibroblast activation by inhibiting multiple tyrosine kinases (Wollin et al., 2015), suggesting that therapeutic approaches targeting fibroblasts are effective to prevent/reverse fibrosis and alleviate pulmonary fibrosis. TGF-β is of greatest interest, as it strongly activates fibroblasts and facilitates ECM production to induce progressive fibrosis in numerous fibrotic diseases (Aschner et al., 2020; Khalil et al., 2017; Derynck and Zhang, 2003). However, TGF-β blockade (e.g., fresolimumab and metelimumab) has major side effects, such as carcinogenesis, systemic autoimmunity, etc.,*.* due to the pleiotropic roles of TGF-β (Henderson et al., 2020). Thus, strategies to avoid these deleterious effects could involve inhibiting TGF-β signaling selectively in excessive activated lung fibroblasts, as TGF-β-triggered Smad2/3 signaling is pivotal in the induction of pulmonary fibrosis in animal models (Figure 6) (Aschner et al., 2020). In this work, we analyzed the effects of CB_1_R-selective agonist ACPA on TGF-β1-challenged lung fibroblasts and found that ACPA markedly suppressed TGF-β1-stimulated activation of TGF-β-Smad2/3 signaling with decreased phosphorylation levels of Smad2 and Smad3 in lung fibroblasts, in addition to reducing the expression of ECM proteins and the transcription factors snail and slug, but hardly having an impact on the expression of ECM proteins in lung fibroblasts under normal conditions. In addition, slug and snail are markers of the epithelial–mesenchymal transition (EMT), which is a process whereby part of the lung epithelial cells, mainly type II alveolar epithelial cells (AT2), transition into cells of the mesenchymal phenotype, such as fibroblasts, or myofibroblasts, and it has been shown to be a critical stage during the development of fibrosis (Nieto et al., 2016). As ACPA inhibited TGF-β1-induced expression of EMT-related transcription factors, slug and snail, and CB_1_R is also found in epithelial cells, including AT2 cells (Cinar et al., 2017), it might be possible that the activation of CB_1_R in the pathogenesis of pulmonary fibrosis also prevented EMT derived from AT2 cells and the ECM it generated. These results revealed that CB_1_R activation, which acted as a negative regulation of canonical TGF-β-Smad2/3 signaling, downregulated TGF-β-triggered overexpression of ECM proteins in lung fibroblasts, but did not affect normal ECM production, and CB_1_R agonist was proven useful as a potential therapeutic option for pulmonary fibrosis. ACPA itself, however, is unlikely to be used in clinical practice because of a short half-life and its lipophilic characteristic to easily enter the central nervous system, while the fibroblast-selective CB_1_R agonist like ACPA nanoparticles and agonistic antibodies to CB_1_R would be promising therapeutic drugs for pulmonary fibrosis that might be used clinically.

In summary, our study showed ACPA, a selective agonist of CB_1_R, which expression increased in pulmonary fibrosis, exhibited antifibrotic efficacy in both *in vitro* and *in vivo* models of pulmonary fibrosis, inhibited TGF-β1-stimulated lung fibroblast activation and fibrotic ECM production *via* downregulating TGF-β1-Smad2/3 signaling, revealing a novel anti-fibrosis approach to fibroblast-selective negative regulation of TGF-β-Smad2/3 signaling by targeting CB_1_R. New candidates selectively targeted to activate CB_1_R on lung fibroblasts might be effective clinical agents for the treatment of pulmonary fibrosis.

## Data Availability

The original contributions presented in the study are included in the article/Supplementary Material, further inquiries can be directed to the corresponding authors.
