# Supplementary material for: ACPA Alleviates Bleomycin-Induced Pulmonary Fibrosis by Inhibiting TGF-β-Smad2/3 Signaling-Mediated Lung Fibroblast Activation
Source: Front Pharmacol. 2022 Mar 9;13:835979. doi: 10.3389/fphar.2022.835979 (PMC8959577; doi:10.3389/fphar.2022.835979)
Supplement: Supplementary file 1 [file Presentation9.PPT]

## Slide 1
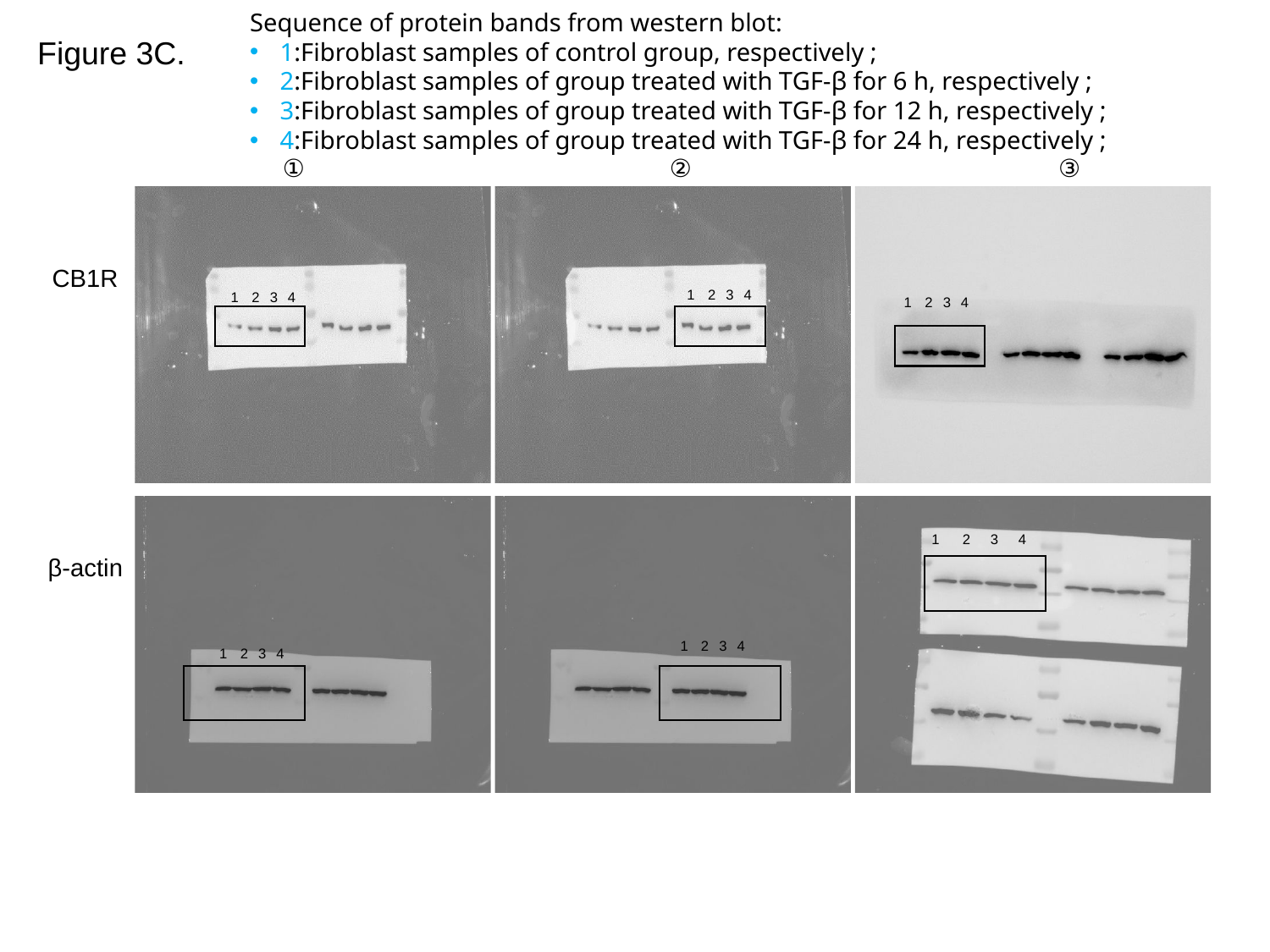

Sequence of protein bands from western blot:
1:Fibroblast samples of control group, respectively ;
2:Fibroblast samples of group treated with TGF-β for 6 h, respectively ;
3:Fibroblast samples of group treated with TGF-β for 12 h, respectively ;
4:Fibroblast samples of group treated with TGF-β for 24 h, respectively ;
Figure 3C.
①
②
③
CB1R
1
2
3
4
1
2
3
4
1
2
3
4
1
2
3
4
β-actin
1
2
3
4
1
2
3
4
