# Supplementary figures and images for: ACPA Alleviates Bleomycin-Induced Pulmonary Fibrosis by Inhibiting TGF-β-Smad2/3 Signaling-Mediated Lung Fibroblast Activation
Source: Front Pharmacol. 2022 Mar 9;13:835979. doi: 10.3389/fphar.2022.835979 (PMC8959577; doi:10.3389/fphar.2022.835979)

## Slide 1
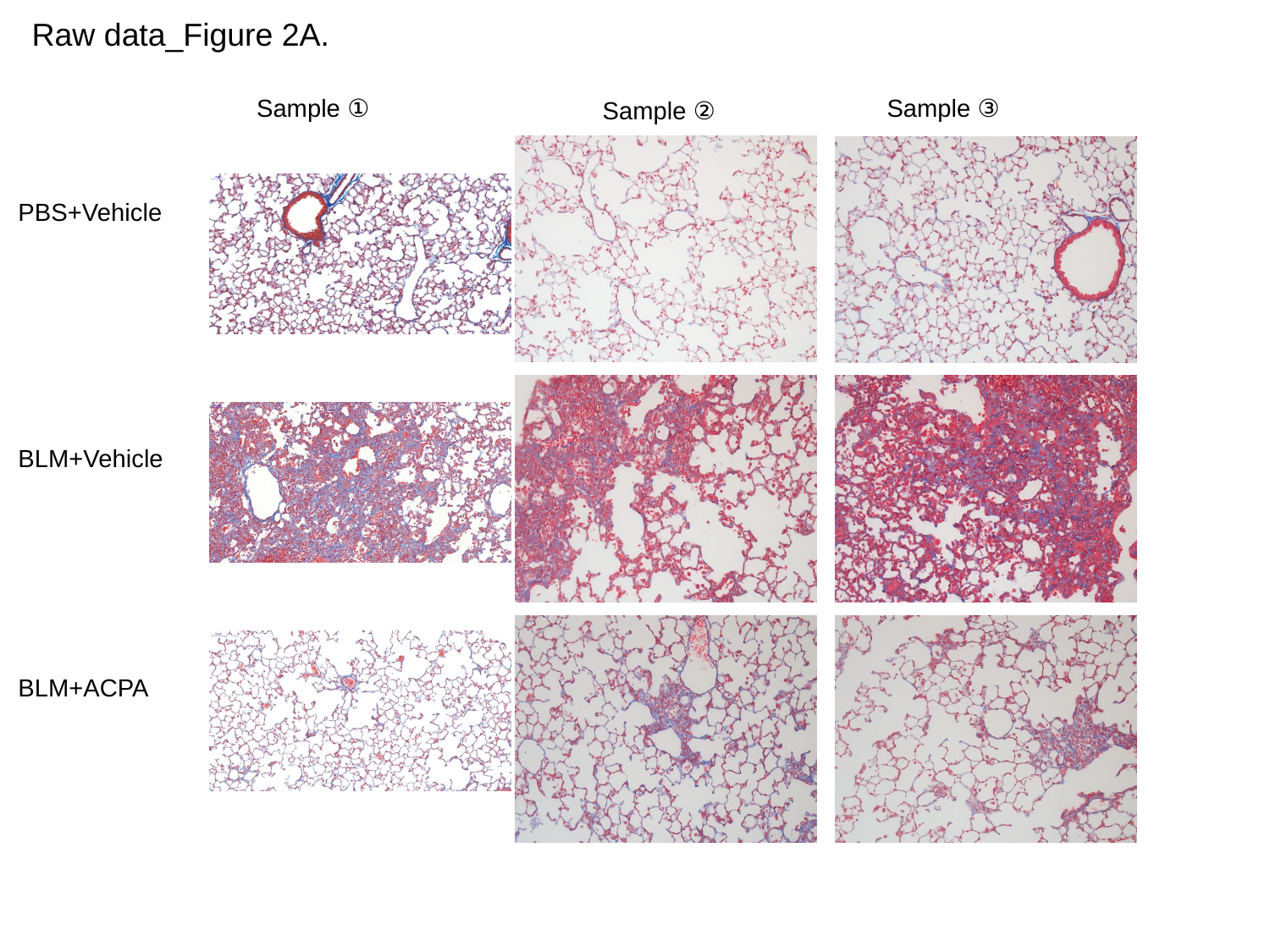

Raw data_Figure 2A.
Sample ①
Sample ③
Sample ②
PBS+Vehicle
BLM+Vehicle
BLM+ACPA

Supplement: Supplementary file 4 [file Presentation2.PPT]

## Slide 1
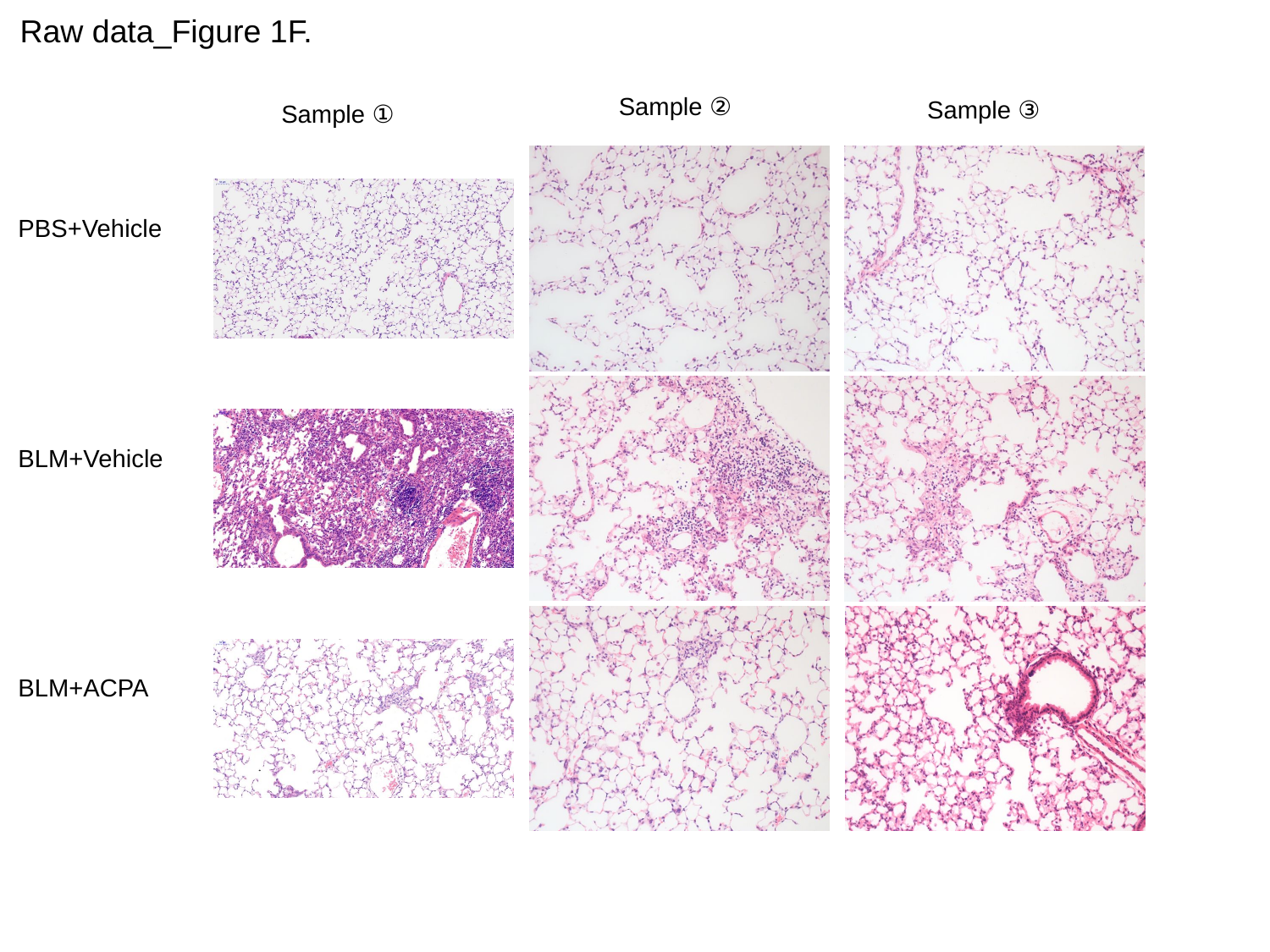

Raw data_Figure 1F.
Sample ②
Sample ③
Sample ①
PBS+Vehicle
BLM+Vehicle
BLM+ACPA

Supplement: Supplementary file 6 [file Presentation1.PPT]

## Slide 1
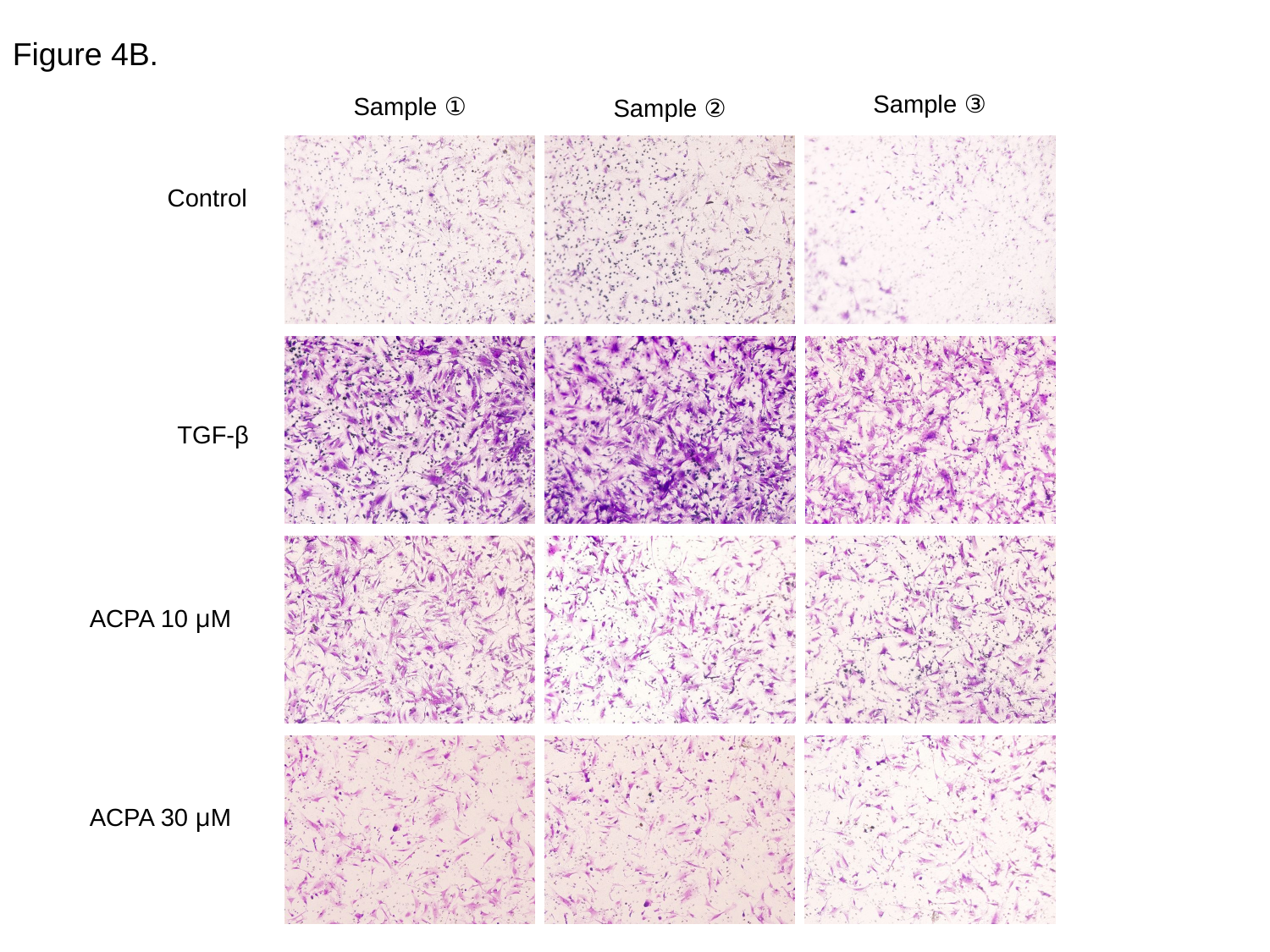

Figure 4B.
Sample ③
Sample ①
Sample ②
Control
TGF-β
ACPA 10 μM
ACPA 30 μM

Supplement: Supplementary file 9 [file Presentation7.PPT]
