# Supplementary material for: ACPA Alleviates Bleomycin-Induced Pulmonary Fibrosis by Inhibiting TGF-β-Smad2/3 Signaling-Mediated Lung Fibroblast Activation
Source: Front Pharmacol. 2022 Mar 9;13:835979. doi: 10.3389/fphar.2022.835979 (PMC8959577; doi:10.3389/fphar.2022.835979)
Supplement: Supplementary file 5 [file Presentation10.PPTX]

## Slide 1
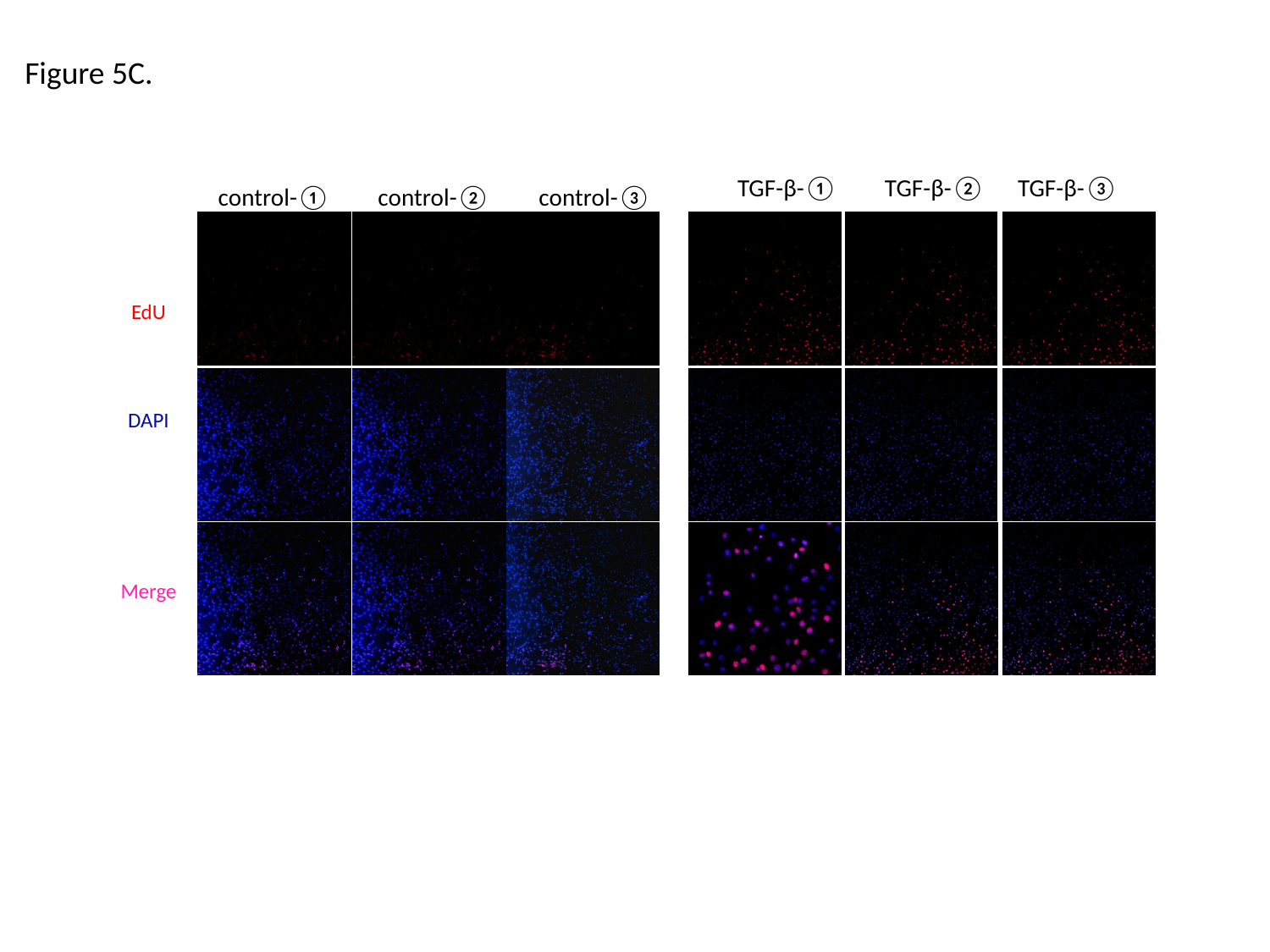

Figure 5C.
TGF-β-①
TGF-β-②
TGF-β-③
control-①
control-②
control-③
EdU
DAPI
Merge

## Slide 2
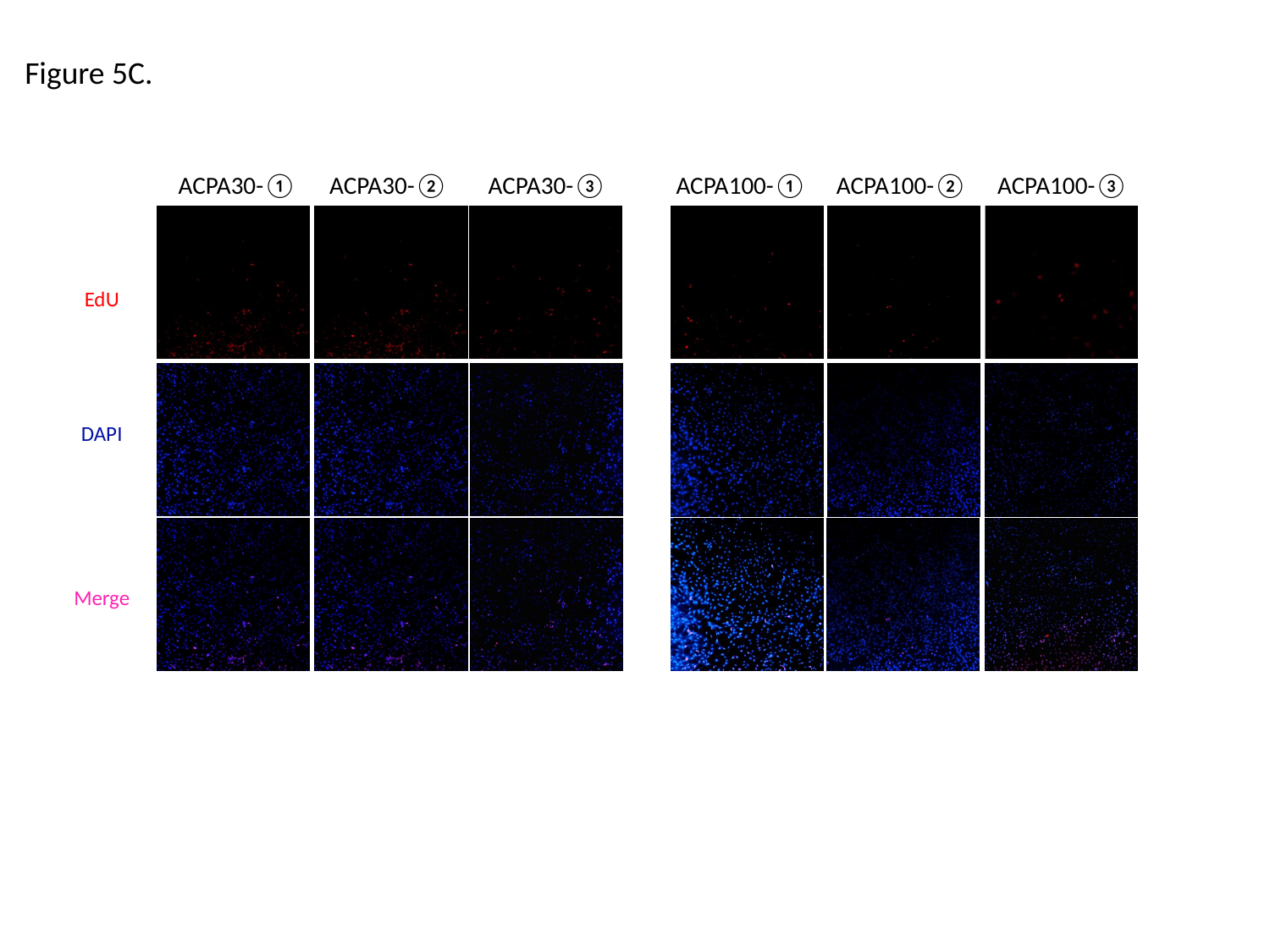

Figure 5C.
ACPA30-①
ACPA30-②
ACPA30-③
ACPA100-①
ACPA100-②
ACPA100-③
EdU
DAPI
Merge
