# Supplementary material for: ACPA Alleviates Bleomycin-Induced Pulmonary Fibrosis by Inhibiting TGF-β-Smad2/3 Signaling-Mediated Lung Fibroblast Activation
Source: Front Pharmacol. 2022 Mar 9;13:835979. doi: 10.3389/fphar.2022.835979 (PMC8959577; doi:10.3389/fphar.2022.835979)
Supplement: Supplementary file 7 [file Presentation3.PPT]

## Slide 1
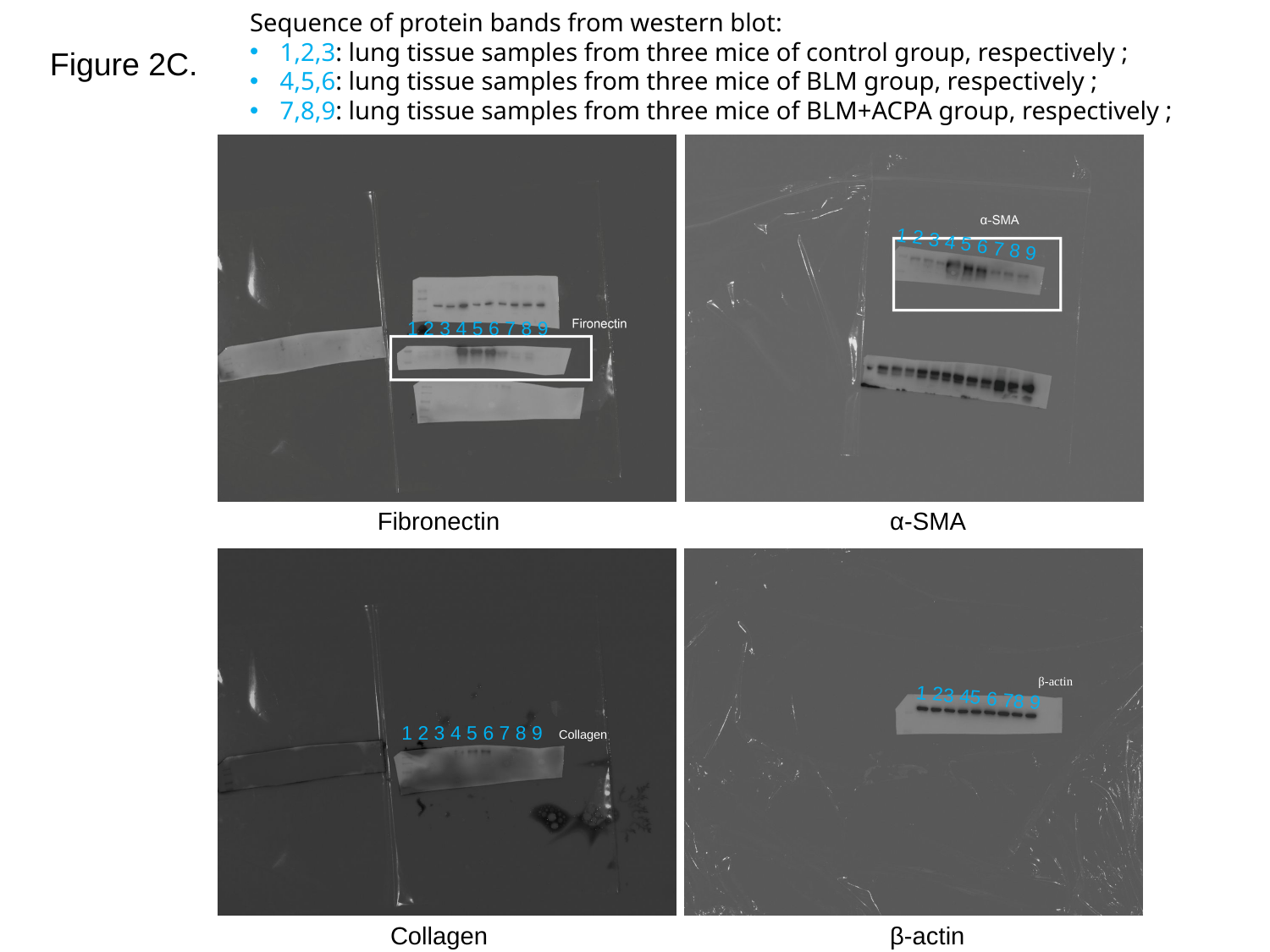

Sequence of protein bands from western blot:
1,2,3: lung tissue samples from three mice of control group, respectively ;
4,5,6: lung tissue samples from three mice of BLM group, respectively ;
7,8,9: lung tissue samples from three mice of BLM+ACPA group, respectively ;
Figure 2C.
1 2 3 4 5 6 7 8 9
1 2 3 4 5 6 7 8 9
Fibronectin
α-SMA
β-actin
1 23 45 6 78 9
1 2 3 4 5 6 7 8 9
Collagen
Collagen
β-actin
