# Supplementary material for: ACPA Alleviates Bleomycin-Induced Pulmonary Fibrosis by Inhibiting TGF-β-Smad2/3 Signaling-Mediated Lung Fibroblast Activation
Source: Front Pharmacol. 2022 Mar 9;13:835979. doi: 10.3389/fphar.2022.835979 (PMC8959577; doi:10.3389/fphar.2022.835979)
Supplement: Supplementary file 8 [file Presentation6.PPT]

## Slide 1
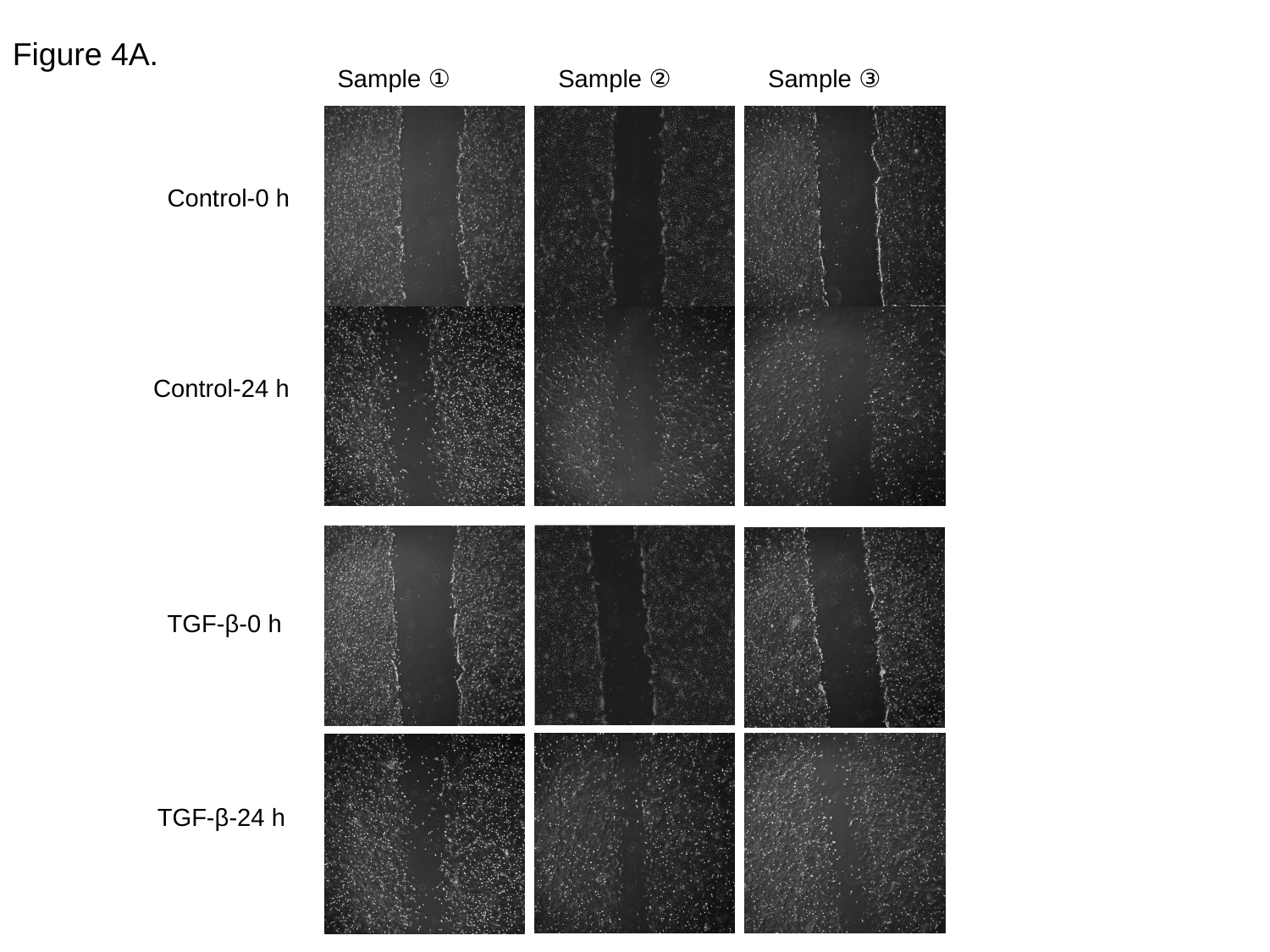

Figure 4A.
Sample ①
Sample ②
Sample ③
Control-0 h
Control-24 h
TGF-β-0 h
TGF-β-24 h

## Slide 2
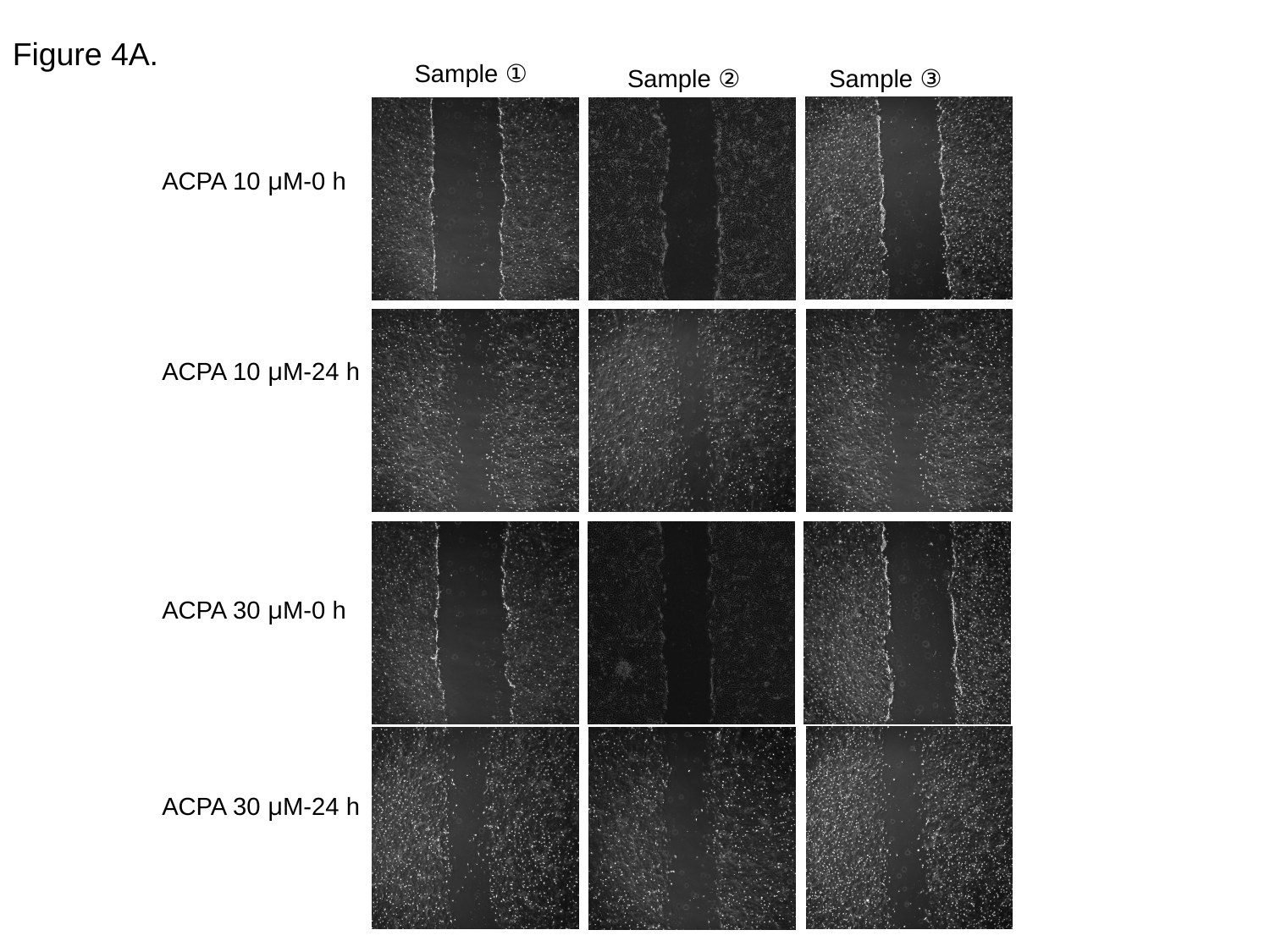

Figure 4A.
Sample ①
Sample ②
Sample ③
ACPA 10 μM-0 h
ACPA 10 μM-24 h
ACPA 30 μM-0 h
ACPA 30 μM-24 h
