# Supplementary material for: ACPA Alleviates Bleomycin-Induced Pulmonary Fibrosis by Inhibiting TGF-β-Smad2/3 Signaling-Mediated Lung Fibroblast Activation
Source: Front Pharmacol. 2022 Mar 9;13:835979. doi: 10.3389/fphar.2022.835979 (PMC8959577; doi:10.3389/fphar.2022.835979)
Supplement: Supplementary file 12 [file Presentation8.PPTX]

## Slide 1
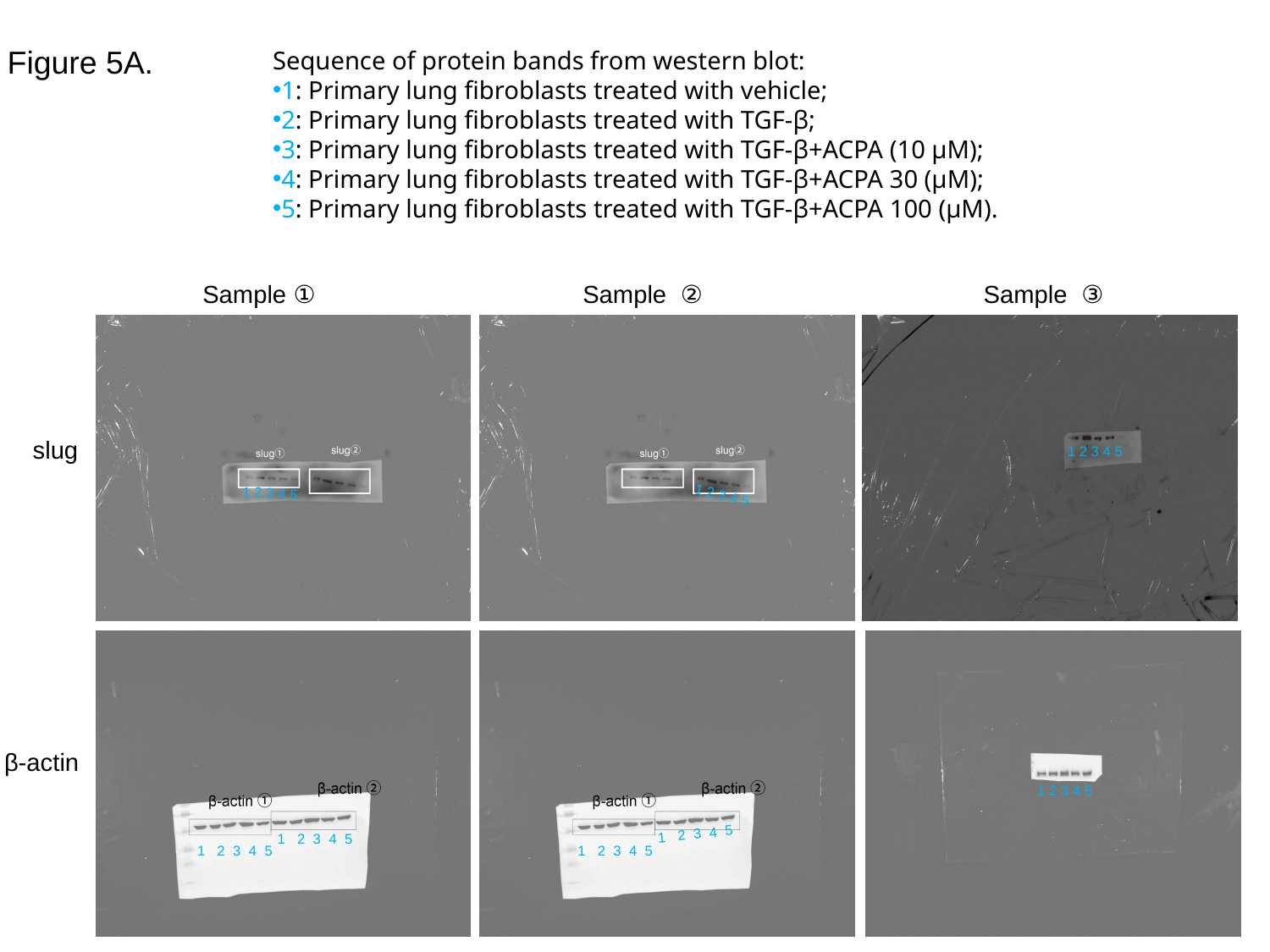

Figure 5A.
Sequence of protein bands from western blot:
1: Primary lung fibroblasts treated with vehicle;
2: Primary lung fibroblasts treated with TGF-β;
3: Primary lung fibroblasts treated with TGF-β+ACPA (10 μM);
4: Primary lung fibroblasts treated with TGF-β+ACPA 30 (μM);
5: Primary lung fibroblasts treated with TGF-β+ACPA 100 (μM).
Sample ①
Sample ②
Sample ③
slug
1 2 3 4 5
1 2 3 4 5
1 2 3 4 5
β-actin
1 2 3 4 5
1 2 3 4 5
1 2 3 4 5
1 2 3 4 5
1 2 3 4 5

## Slide 2
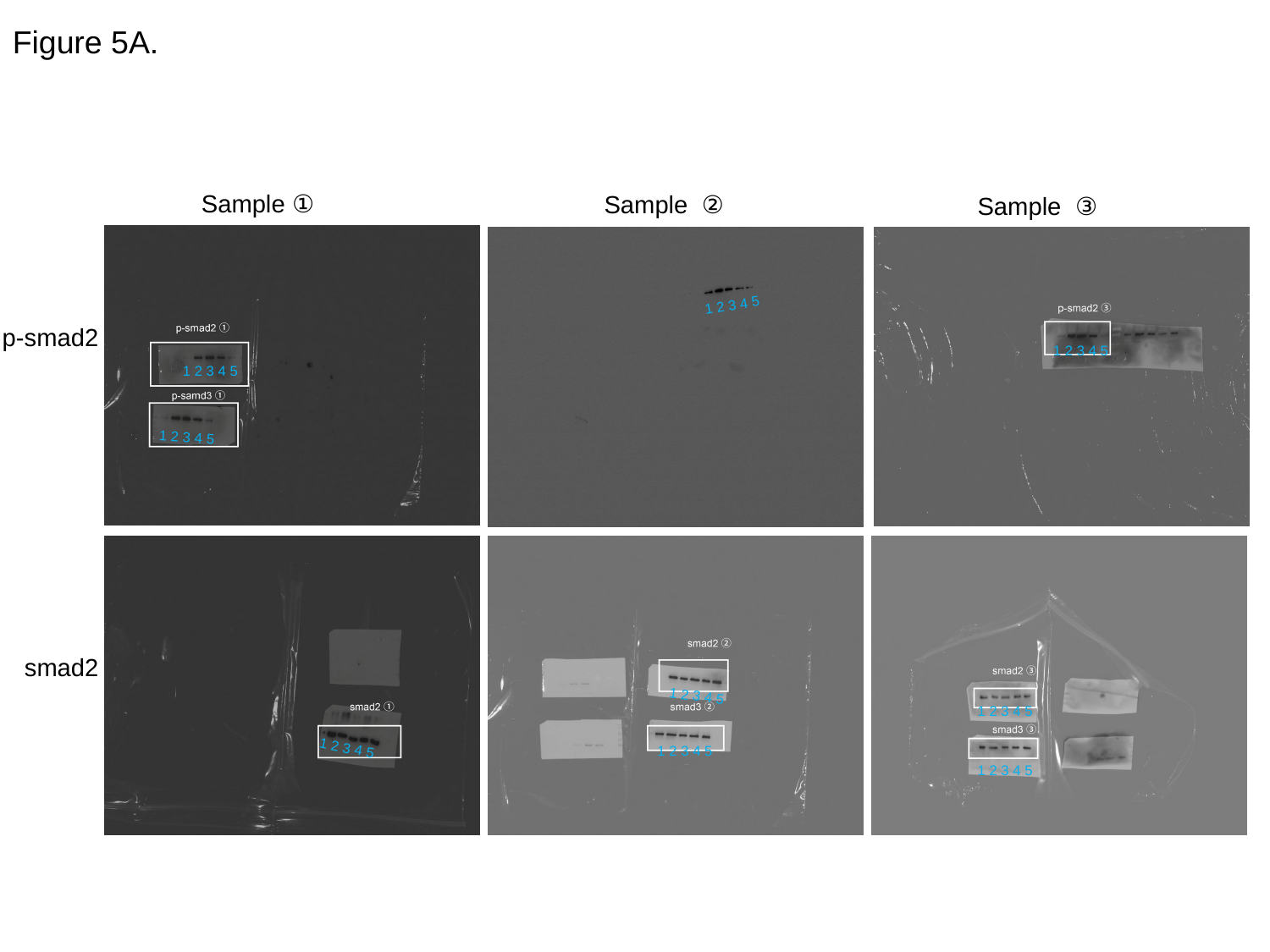

Figure 5A.
Sample ①
Sample ②
Sample ③
1 2 3 4 5
p-smad2
1 2 3 4 5
1 2 3 4 5
1 2 3 4 5
smad2
1 2 3 4 5
1 2 3 4 5
1 2 3 4 5
1 2 3 4 5
1 2 3 4 5

## Slide 3
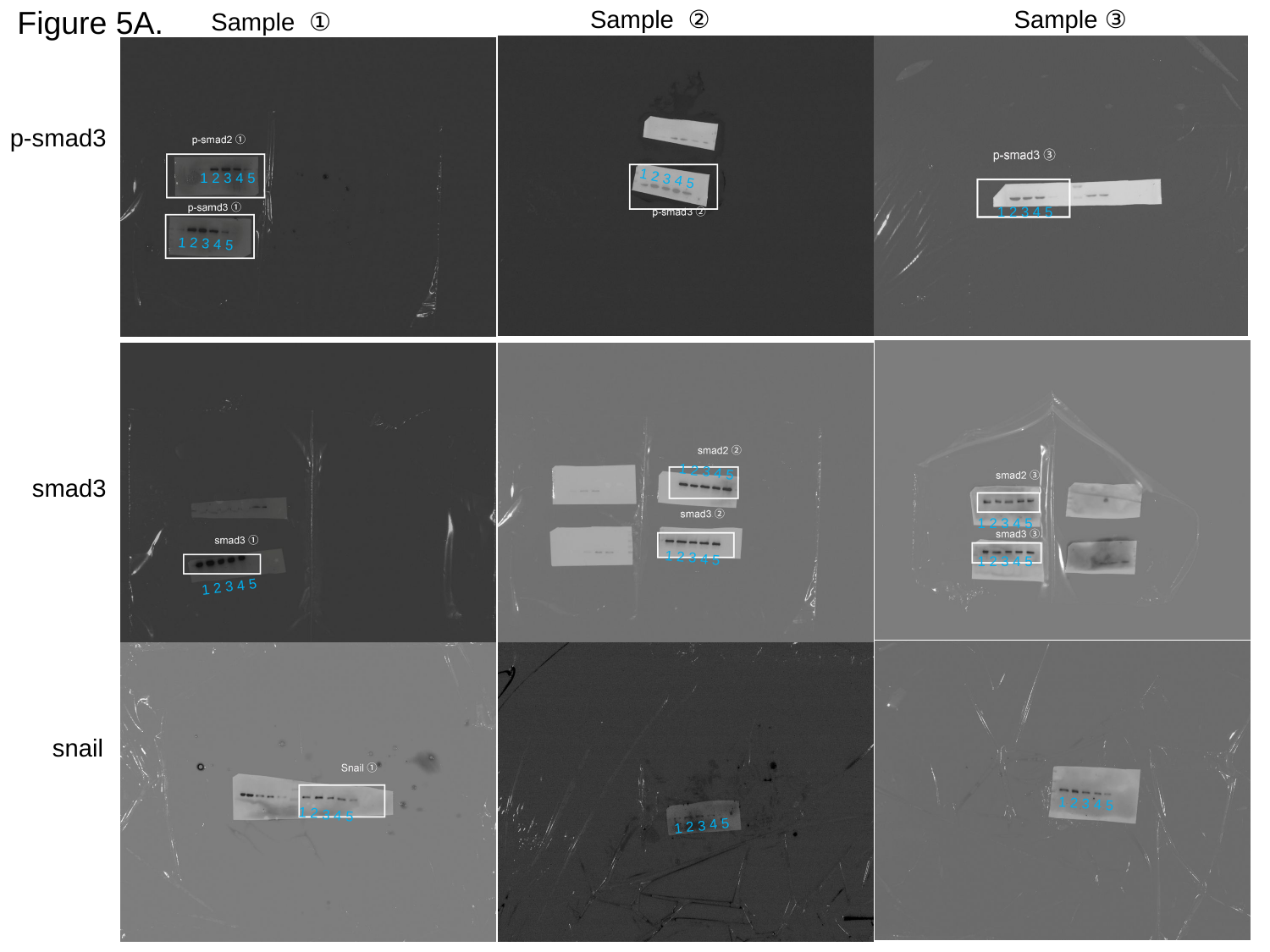

Sample ①
Figure 5A.
Sample ②
Sample ③
p-smad3
1 2 3 4 5
1 2 3 4 5
1 2 3 4 5
1 2 3 4 5
1 2 3 4 5
smad3
1 2 3 4 5
1 2 3 4 5
1 2 3 4 5
1 2 3 4 5
snail
1 2 3 4 5
1 2 3 4 5
1 2 3 4 5
