# Supplementary material for: ACPA Alleviates Bleomycin-Induced Pulmonary Fibrosis by Inhibiting TGF-β-Smad2/3 Signaling-Mediated Lung Fibroblast Activation
Source: Front Pharmacol. 2022 Mar 9;13:835979. doi: 10.3389/fphar.2022.835979 (PMC8959577; doi:10.3389/fphar.2022.835979)
Supplement: Supplementary file 13 [file Presentation5.PPTX]

## Slide 1
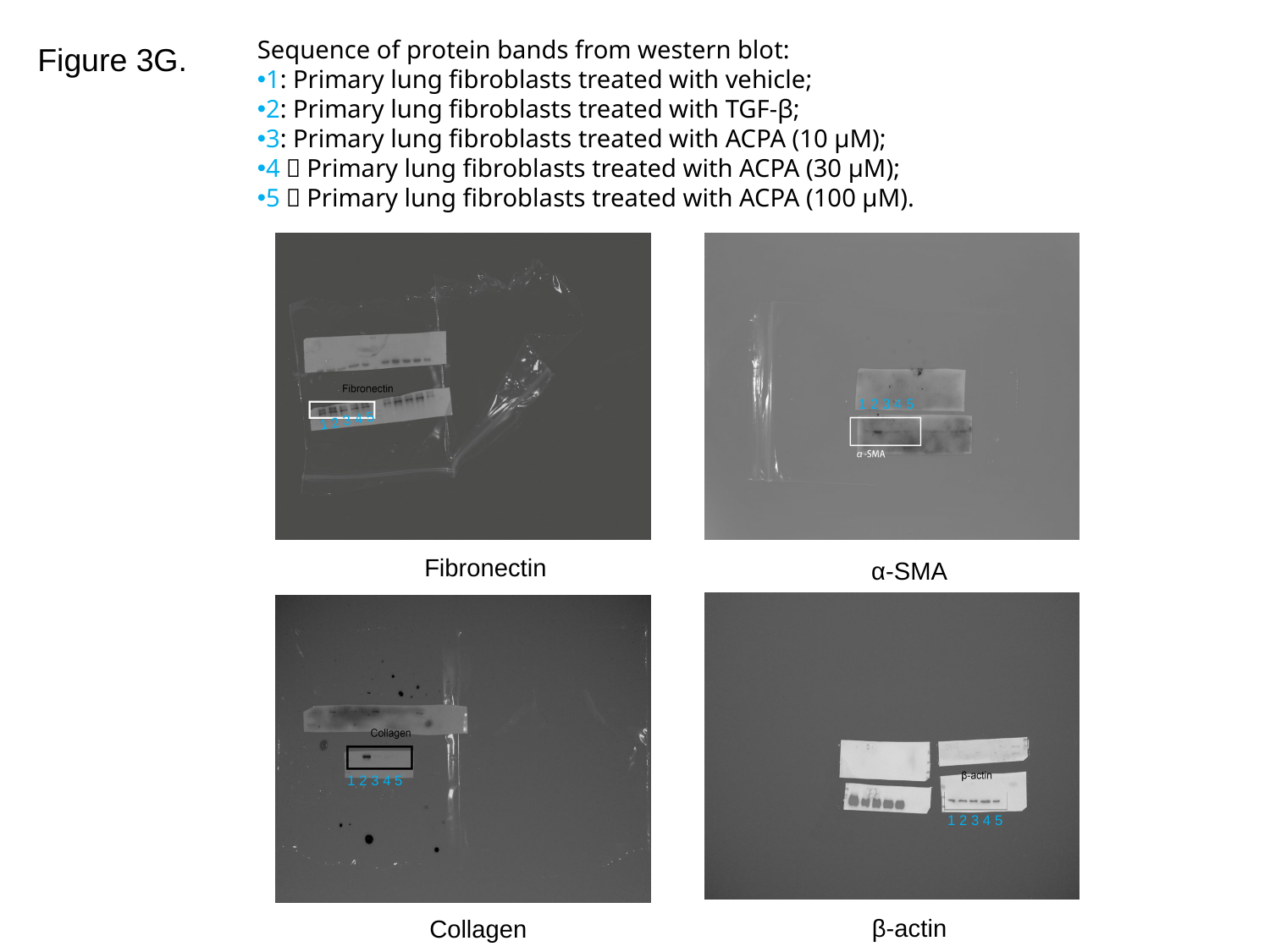

Sequence of protein bands from western blot:
1: Primary lung fibroblasts treated with vehicle;
2: Primary lung fibroblasts treated with TGF-β;
3: Primary lung fibroblasts treated with ACPA (10 μM);
4：Primary lung fibroblasts treated with ACPA (30 μM);
5：Primary lung fibroblasts treated with ACPA (100 μM).
Figure 3G.
1 2 3 4 5
1 2 3 4 5
Fibronectin
α-SMA
1 2 3 4 5
1 2 3 4 5
β-actin
Collagen
